# Multivalent Presentations of Glycomimetic Inhibitor of the Adhesion of Fungal Pathogen *Candida albicans* to Human Buccal Epithelial Cells

**DOI:** 10.1021/acs.bioconjchem.1c00115

**Published:** 2021-04-22

**Authors:** Harlei Martin, David Goyard, Anatte Margalit, Kyle Doherty, Olivier Renaudet, Kevin Kavanagh, Trinidad Velasco-Torrijos

**Affiliations:** † Department of Chemistry, Maynooth University, Maynooth, W23VP22, Co. Kildare, Ireland; ‡ DCM, UMR 5250, Université Grenoble Alpes, CNRS, 38000 Grenoble, France; § Department of Biology, Maynooth University, Maynooth, W23VP22, Co. Kildare, Ireland; ∥ The Kathleen Lonsdale Institute for Human Health Research, Maynooth University, Maynooth, W23VP22, Co. Kildare, Ireland

## Abstract

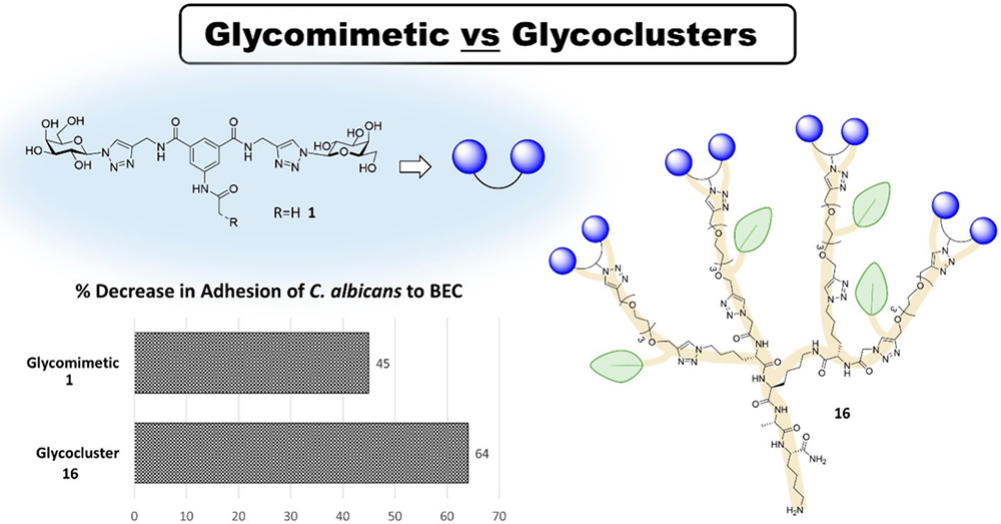

*Candida albicans* causes some of the most prevalent hospital-acquired fungal infections, particularly threatening for immunocompromised patients. *C. albicans* strongly adheres to the surface of epithelial cells so that subsequent colonization and biofilm formation can take place. Divalent galactoside glycomimetic **1** was found to be a potent inhibitor of the adhesion of *C. albicans* to buccal epithelial cells. In this work, we explore the effect of multivalent presentations of glycomimetic **1** on its ability to inhibit yeast adhesion and biofilm formation. Tetra-, hexa-, and hexadecavalent displays of compound **1** were built on RAFT cyclopeptide- and polylysine-based scaffolds with a highly efficient and modular synthesis. Biological evaluation revealed that the scaffold choice significantly influences the activity of the lower valency conjugates, with compound **16**, constructed on a tetravalent polylysine scaffold, found to inhibit the adhesion of *C. albicans* to human buccal epithelial cells more effectively than the glycomimetic **1**; however, the latter performed better in the biofilm reduction assays. Interestingly, the higher valency glycoconjugates did not outperform the anti-adhesion activity of the original compound **1**, and no significant effect of the core scaffold could be appreciated. SEM images of *C. albicans* cells treated with compounds **1**, **14**, and **16** revealed significant differences in the aggregation patterns of the yeast cells.

## Introduction

The initiation of a multitude of human diseases is mediated by protein–carbohydrate recognition.^1,2^ For a microbe to infect its host, it first adheres to the host cell using adhesion proteins, many of which recognize carbohydrate epitopes displayed on the host cell surface.^3^ The development of small-molecule inhibitors of this adhesion process has been extensively studied. Glycoconjugates that mimic the glycans displayed on host cell surfaces are attractive candidates for the development of anti-adhesion compounds.^4^ Numerous ligands for microbial lectins found in pathogens such as *Pseudomonas aeruginosa*, *Aspergillus fumigatus*, and *Escherichia coli* have been reported.^5−10^ Significantly, the crystal structures of these adhesins have been solved, which has facilitated structure-based ligand design.^11−13^ However, carbohydrates interact with their protein receptors with low affinity, with dissociation constants typically in the millimolar to micromolar range.^14^ Consequently, a major focus of glycoscience research involves the development of strategies for increasing the lectin-ligand binding affinities to levels required for therapeutic use.^15^ This can be achieved through the glycomimetic approach or through utilizing the multivalent or cluster glycoside effect.^5,16^ Multivalent glycoconjugates with diverse valencies and spatial arrangements of recognition epitopes have been developed to increase the affinity of carbohydrates for their target proteins.^17^ These multivalent constructs can have well-defined molecular structures and display a specific number of carbohydrate ligands when they are built around scaffolds such as calixarenes,^18^ dendrimers,^19^ cyclodextrins,^20^ and fullerenes;^21^ higher valencies with more variable degrees of substitution are achieved when using polymers,^22^ nanoparticles,^23^ and quantum dots.^24^ Even with the extensive research carried out in this field, it still remains extremely challenging to predict optimal multivalent presentations that result in enhanced binding affinities for a specific target.

Lysine-containing cyclodecapeptides called “regioselectively addressable functionalized templates” (RAFT) were first described as stable scaffolds for the *de novo* design of proteins or as peptidomimics.^25^ These scaffolds have a defined and constrained structure, and the presence of the lysine residue in the amino acid sequence has allowed for further functionalization and display of carbohydrates in a multivalent manner. In order to improve the recognition properties of the cyclopeptide-based glycoclusters toward lectins, newer generations with higher valency and varying levels of rigidity have been developed by Renaudet and co-workers. A modular chemoselective strategy was used to introduce either a flexible polylysine framework or a constrained cyclopeptide onto the RAFT core, providing two different hyperbranched skeletons in a controlled manner. Biologically relevant carbohydrates were then conjugated to obtain a new series of glycodendrimer-like structures.^26^ These structures have been used to generate highly potent ligands for interactions with different lectins.^27^ For example, multivalent cyclopeptide scaffolds presenting *N*-acetyl glucosamine (GlcNAc), *N*-acetyl galactosamine (GalNAc), and, more recently, mannose (Man) moieties have been shown to bind the lectins wheat germ agglutinin,^28^ soybean agglutinin,^29^ and DC-SIGN,^30^ respectively, at micro- and nanomolar concentrations. Glycoclusters based on RAFT and polylysine scaffolds displaying several copies of α-fucose (Fuc) and β-galactose (Gal) have also been found to have high affinity for LecA and LecB, important adhesins from *Pseudomonas aeruginosa*.^31,32^ A glycodendron displaying 24 α-mannosides was found to bind BC2L-A, a lectin from respiratory pathogen *Burkholderia cenocepacia*, at nanomolar concentration.^33^ Interestingly, glycomimetics with affinity toward fucose-binding receptors from emerging pathogens *Aspergillus fumigatus* and *Burkholderia ambifaria* were grafted onto hexavalent RAFT scaffolds and showed dissociation constants in the low nanomolar range.^34^

**Figure 1:**
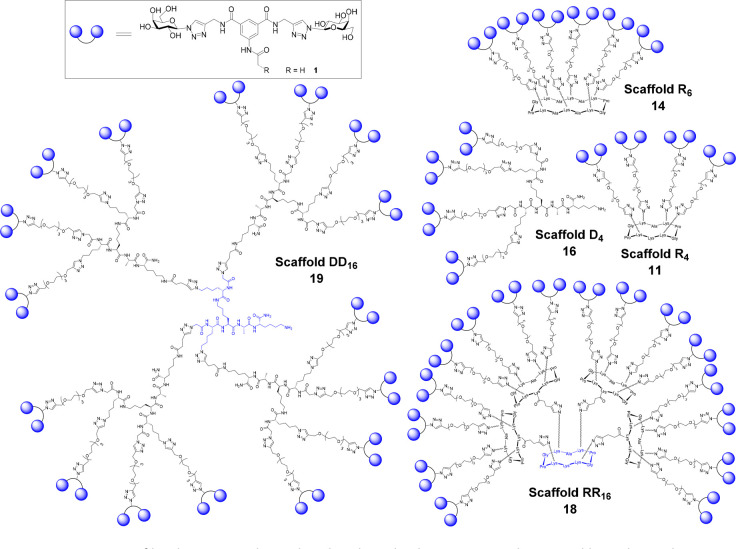
Structure of lead compound **1** and multivalent displays on tetra-, hexa-, and hexadecavalent RAFT cyclopeptides (R_4_**11**, R_6_**14**, RR_16_**18**) and dendrimeric polylysine (D_4_**16**, DD_16_**19**) scaffolds.

*Candida albicans* is an opportunistic pathogenic yeast and is the most prevalent cause of fungal infections worldwide, particularly in hospital-acquired infections.^35−37^ The yeast is capable of causing a wide range of superficial and systemic infections in the immunocompromised patient, and there is now also growing concern for the coinfection of fungal pathogens in Covid-19 patients.^38−40^ It is therefore extremely important that new treatments are developed for these fungal infections, some of which are now showing resistance to conventional antifungal therapies.^41^ Compound **1** (Figure 1) was previously reported by us as a successful inhibitor of *C. albicans* adhesion to human buccal epithelial cells (BECs).^42,43^ The presence of the two triazolyl galactosides in compound **1** was found to be critical for its activity, as glycoconjugates with carbohydrates other than galactose displayed on the same aromatic scaffold had reduced the anti-adhesion effect or provided no effect at all. Given the versatility of the RAFT cyclopeptide and dendrimeric polylysine scaffolds, herein we describe their use to prepare multivalent displays of the glycomimetic **1** (shown in Figure 1) and investigate their efficacy at inhibiting the adhesion of *C. albicans* to BECs. Lead compound **1** was modified with a suitable triethylene glycol linker to facilitate connection with these scaffolds using the highly efficient copper-catalyzed azide–alkyne cycloaddition (CuAAC) methodology. Since the structure of the target lectin in *C. albicans* is not known, ligand optimization requires the screening of a diverse range of multivalent presentations to identify the optimum structural parameters (i.e., valency, flexibility, and three-dimensional orientation) to achieve increased anti-adhesion activities.

## Results and Discussion

### Chemical Synthesis

**Scheme 1:**
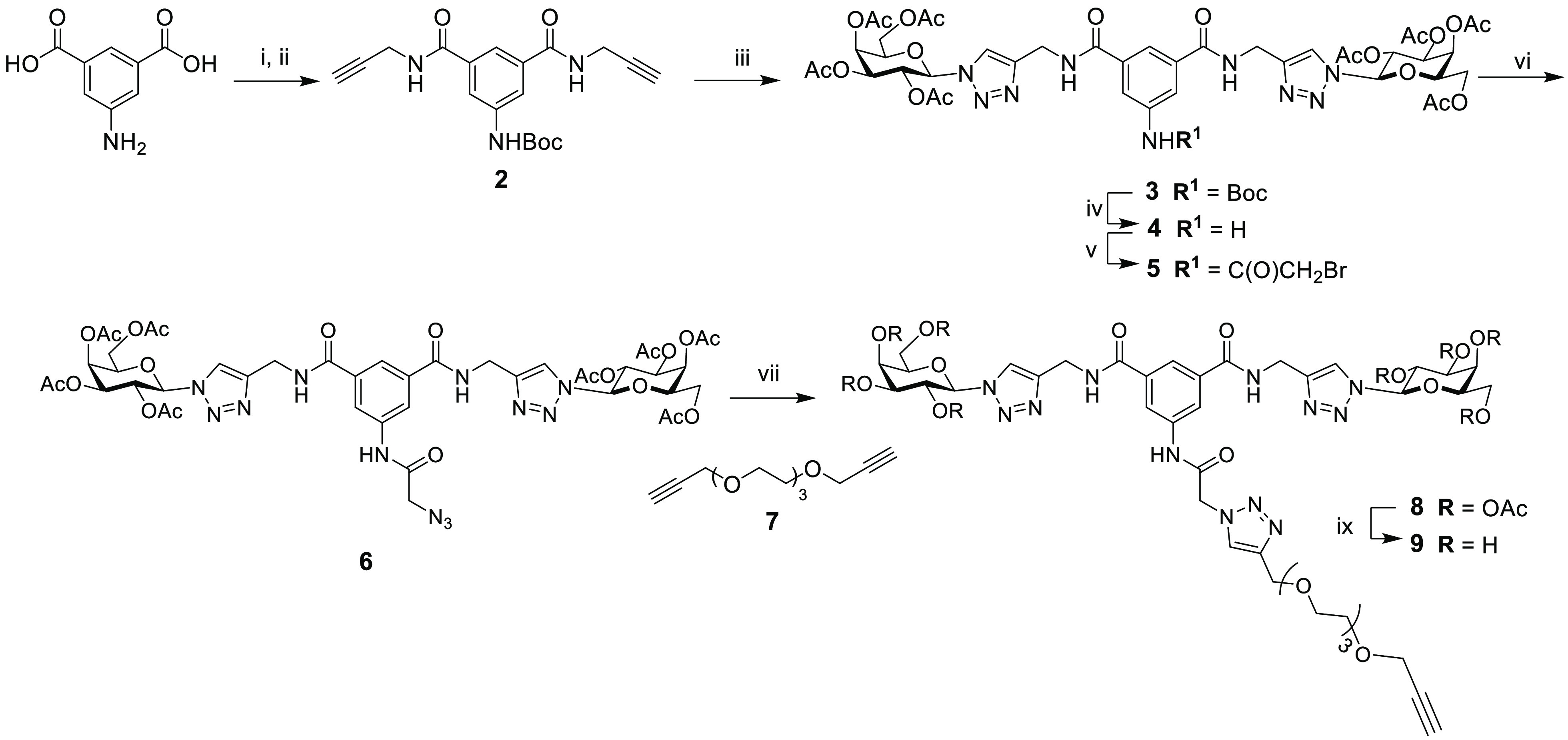
Inset Shows the Structure of Lead Compound **1**. Synthesis of Compound **9** Reagents and conditions: (i) Di-*tert*-butyl dicarbonate, NaOH, 1,4-dioxane, 0 °C to rt, 3 h, 86%; (ii) DMTMM, propargylamine, THF, 48 h, 95%; (iii) 2,3,4,6-tetra-*O*-acetyl-1-β-azido-galactoside, CuSO_4_·5H_2_O/sodium ascorbate (Na Asc), CH_3_COCH_3_/H_2_O, rt, 16 h, 71%; (iv) TFA, DCM, 2 h, rt, 99%; (v) bromoacetyl bromide, NEt_3_, anhydrous DCM, 16 h, 83%; (vi) NaN_3_, anhydrous DMF, N_2_, 80 °C, 16 h, quant %; (vii) **7**, CuSO_4_·5H_2_O/Na Asc, CH_3_CN/H_2_O, MW, 100 °C, 10 min, 45%; (ix) MeOH, NEt_3_, H_2_O, 45 °C, 6 h, 90%.

The scaffolds chosen for this study included RAFT cyclopeptides containing four (R_4_, **10**) or six (R_6_, **13**) lysines oriented out of the upper domain of the scaffold, and a flexible lysine-based dendron (D_4_, **15**), all previously reported by Renaudet and co-workers.^32,33,44^ All these scaffolds featured azide groups, which required the functionalization of lead compound **1** with an alkyne group in order to use CuAAC protocols. Lead compound **1** was therefore modified with a triethylene glycol linker with a terminal propargyl group as shown in Scheme 1. Based on our previously reported synthesis,^42^ 5-amino-isophthalic acid was *N*-Boc protected, followed by reaction with propargylamine and TBTU to give aromatic-centered diamide scaffold **2**. CuAAC chemistry was used to conjugate the acetylated galactose azides to the scaffold to give compound **3**. *N*-Boc-deprotection with TFA yielded compound **4**, which was reacted with bromoacetyl bromide to give **5** and subsequently converted to compound **6** by reaction with sodium azide. Separately, triethylene glycol was reacted with propargyl bromide to give dialkyne linker **7**.^45^ This was then reacted with the azide derivative **6** using CuAAC methodology. To promote reaction with only one alkynyl group, this reaction was carried out in dilute conditions and with a large excess of linker **7**, giving the desired monoalkynyl compound **8** and unreacted linker **7** as the major outcomes of the reaction after column chromatography. Deacetylation of **8** under mild basic conditions gave key synthetic intermediate **9**, as it presents an alkyne that will allow for conjugation to the peptidic scaffold functionalized with azido groups.

**Scheme 2:**
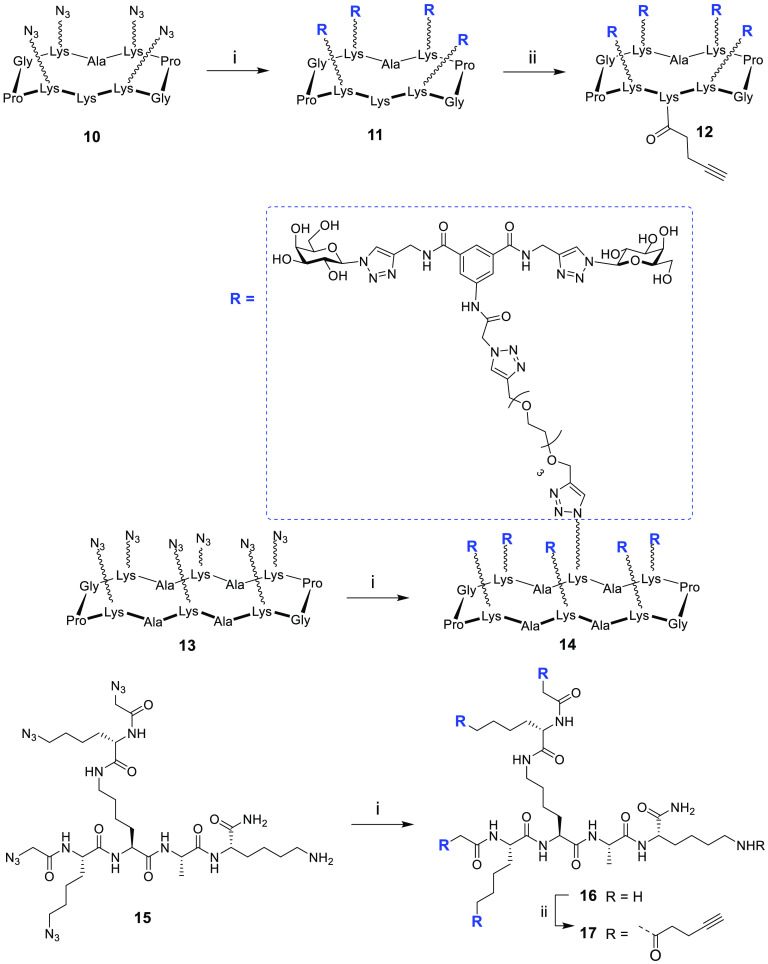
Synthesis of Glycoclusters Reagents and conditions: (i) **9**, CuSO_4_·5H_2_O/Na Asc, THPTA, DMF/PBS buffer (pH 7.5), rt, 1 h, 34–78%; (ii) *N*-succinimidyl pentynoate, DIPEA, DMF (pH 9), rt, 1 h, 82–97%. All amino acids have l-configuration.

We next prepared three different glycoclusters with valencies of four and six. Azide-containing cyclopeptide scaffolds R_4_**10** and R_6_**13** and lysine-based dendron D_4_**15** were prepared using previously reported procedures.^32,33,44^ Coupling of these three scaffolds with compound **9** using the CuAAC protocol gave glycoclusters **11**, **14**, and **16**, respectively. In order to prepare glycodendrimers with higher valencies, compounds **11** and **16** were further functionalized by reacting them with *N*-succinimidyl pentynoate under basic conditions to yield compounds **12** and **17**, respectively (Scheme 2).

**Scheme 3:**
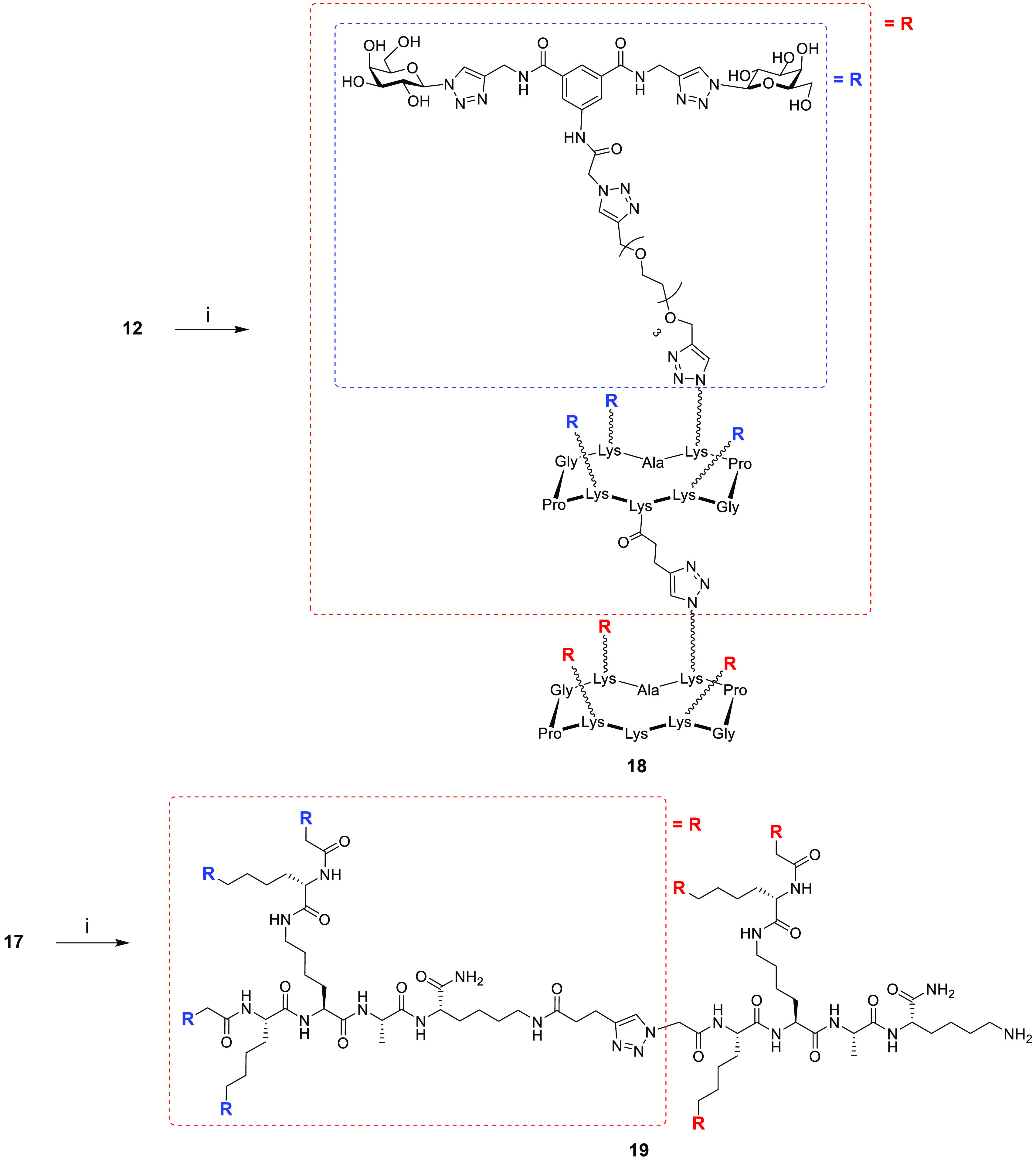
Synthesis of Glycodendrimers **18** and **19** Reagents and conditions: (i) CuSO_4_·5H_2_O/Na Asc, THPTA, DMF/PBS buffer (pH 7.5), rt, 1 h, 87–89%.

Compound **12** was then coupled to another azide-functionalized RAFT cyclopeptide **10** using the same CuAAC conditions to give glycocluster **18**. This compound features 16 copies of the lead compound attached to rigid cyclopeptide cores, resulting in the display of 32 galactose residues. Similarly, compound **17** was then coupled to another azide-functionalized polylysine scaffold **15** to give the glycodendrimer **19**, which, like compound **18**, has 16 copies of the lead compound. However, compound **19** provides a more flexible presentation of the 32 galactose moieties (Scheme 3). All the final compounds were purified by preparative HPLC and characterized by mass spectrometry.

### Biological Evaluation

**Figure 2:**
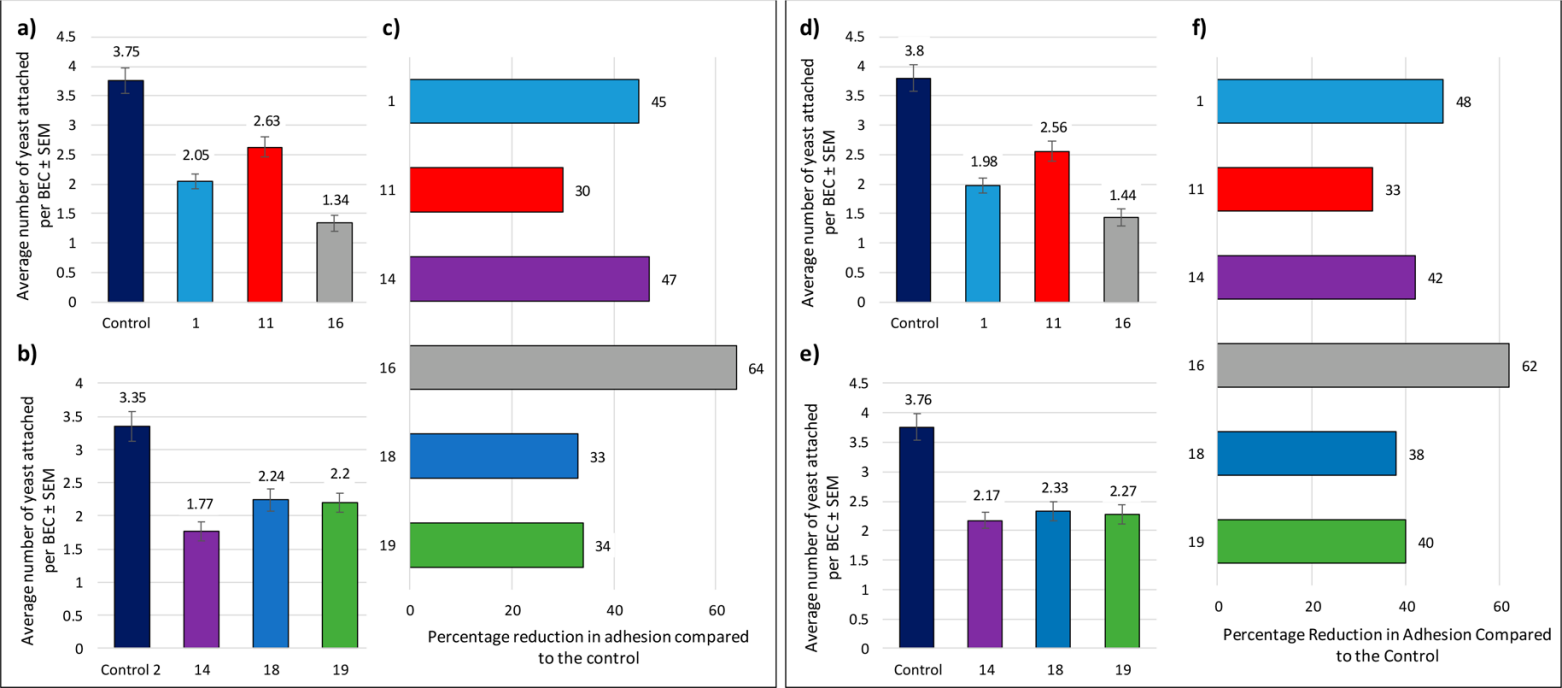
Left: Exclusion assays where *C. albicans* yeast cells were pretreated with the glycoconjugates before incubation with BECs: (a) and (b) show the average number of yeast cells attached per BEC; (c) shows the percentage reduction in adhesion compared to the control (Phosphate Buffer solution, PBS). Right: Competition assays where the *C. albicans* yeast cells, BECs, and glycoconjugates were coincubated: (a) and (b) show the average number of yeast cells attached per BEC; (c) shows the percentage reduction in adhesion compared to the control (PBS). All compounds were tested at 1 mg/mL (see also ESI—Table 1).

Adhesion assays to assess the ability of the multivalent compounds to inhibit *C. albicans* adhesion to human BECs were carried out with all glycoclusters and dendrimers, using the original lead compound **1** as a positive control (Figure 2). An exclusion assay was first performed where the yeast cells were pretreated with the glycoconjugates, prior to exposure to BECs. As seen in Figure 2, the tetravalent polylysine glycodendrimer **16** achieved better results than lead compound **1**, inhibiting the yeast adhesion by 64%. Interestingly, tetravalent glycocuster **11**, based on a RAFT cyclopeptide scaffold, showed only 30% inhibition, approximately half of the activity of analogue **16**, highlighting the critical role of the core scaffolds on the overall presentation of the recognition epitopes. Compound **14**, based on a RAFT hexavalent scaffold, had very comparable results to the original compound **1** (47% and 45%, respectively). On the other hand, the higher valency glycoconjugates **18** and **19**, displaying 16 copies of the lead compound **1**, performed very similarly, independently of the structure of the core scaffolds used: neither of them surpassed the inhibition activity of compound **1**, with 33% inhibition for RAFT-based **18** and 34% inhibition for glycodendrimer **19**.

The competition assays, where *C. albicans* yeast cells, BECs, and glycoclusters/dendrimers were coincubated, showed a similar trend to the previous assays (Figure 2): Again, compound **16** based on the tetravalent polylysine scaffold was the most effective inhibitor, with a 62% reduction of adhesion, outperforming lead compound **1** (48%). Both tetra- and hexavalent glycoconjugates built on cyclic RAFT scaffolds (compounds **11** (33%) and **14** (42%), respectively) did not inhibit the adhesion as well as compound **1.** The higher valencies displayed by both the hexadecavalent glycoconjugates **18** (38%) and **19** (40%) resulted once again in comparable inhibition of adhesion of *C. albicans*, irrespectively of the core scaffold architectures.

### SEM Imaging

**Figure 3:**
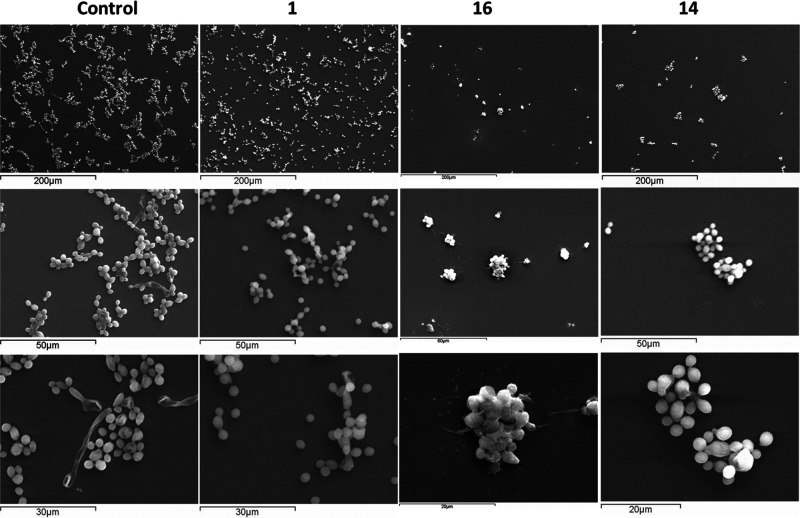
SEM images of *C. albicans* yeast cells at different magnifications after treatment with PBS, lead compound **1**, tetravalent glycodendrimer **16**, and hexavalent glycocluster **14**.

SEM was used to observe the morphology of *C. albicans* cells after 24 h exposure to the lead compound **1** and glycoclusters **14** and **16**, the best performing of the multivalent glycoconjugates (Figure 3). In the control (not treated) samples, the transition of some of the yeast cells to hyphae and pseudohyphae can be observed, while these morphologies were not evident in the samples treated with the glycoconjugates. Interestingly, while larger clumps of yeast cells were observed for the control sample, the cells treated with compound **1** appeared more dispersed. The yeast exposed to glycodendrimers **14** and **16** showed just a few cells aggregating tightly. These images suggest that, although both compound **1** and **16** display the same divalent galactoside glycomimetic epitope, the mechanisms by which they interact with structural components of the cell wall in *C. albicans* may be different. Yeast adhesion processes are critical in the development of biofilms.^46^ The ability of these compounds to interfere with adhesion processes may be advantageous to prevent morphogenesis and biofilm formation, which are major virulence factors in *C. albicans* infections.^47^

### Biofilm Assays

Biofilm assays were then carried out using d-galactose, lead compound **1**, and multivalent glycodendrimer **16**. *C. albicans* has the ability to form biofilms, a major virulence characteristic of this pathogen.^48^ The extracellular matrix which forms during the maturation stage of biofilm development, encompasses a complex network of yeast, pseudohyphal, and hyphal cells, forming densely packed groups of cells adhered to a surface. These biofilms are inherently resistant to conventional antifungal drugs and the host immune system, causing a huge problem in clinical infections.^49^ Hence, the discovery of a treatment that can inhibit the initial adhesion process of the yeast and prevent the formation of biofilms is hugely desirable.

**Figure 4:**
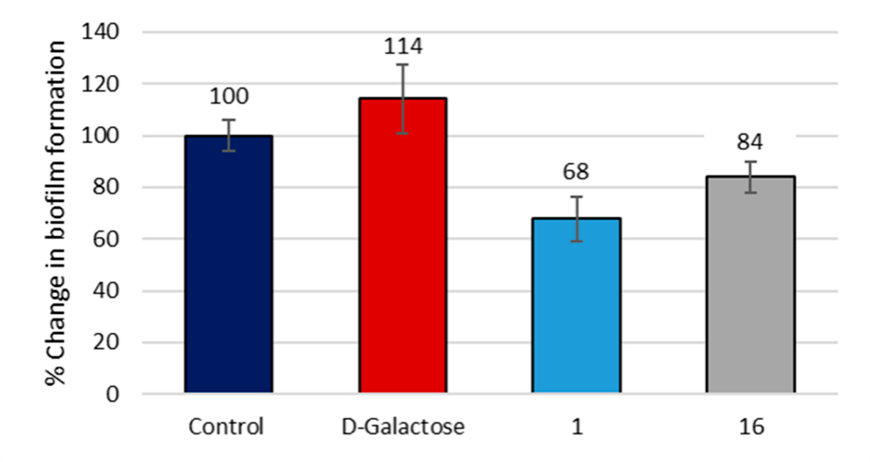
Biofilm inhibition showing the percentage change in the biofilm formation. Control: PBS. (Error: Standard deviation).

Before the biofilm assays, the compounds were tested for their fungicidal properties, showing no toxicity toward *C. albicans* (Figure S24). Under biofilm growing conditions, *C. albicans* cultures were treated with d-galactose as a positive control,^50^ lead compound **1** and glycodendrimer **16**. As shown in Figure 4, d-galactose promoted the formation of the biofilm, while glycomimetic **1** decreased the formation of the biofilm by over 30%, highlighting the potential antivirulence activity of this compound. Surprisingly, even though compound **16** preformed best in the adhesion assays, it was slightly less effective than **1** at reducing biofilm formation (26%).

In summary, five novel multivalent displays of anti-adhesion glycomimetic **1** were successfully synthesized. The core divalent galactosyl structure required for biological activity was functionalized with a triethylene glycol linker appended with a terminal alkyne group. Using CuAAC chemistry, this was connected to azide-containing scaffolds to form tetra-, hexa-, and hexadecavalent RAFT cyclopeptides (R_4_**11**, R_6_**14**, RR_16_**18**) and dendrimeric polylysine (D_4_**16**, DD_16_**19**) based multivalent displays of compound **1**. Anti-adhesion assays on these compounds (Table 1) showed that the core peptidic scaffold strongly influences the presentation of the glycomimetic structure in the multivalent glycoclusters, and hence their ability to inhibit adhesion of *C. albicans* to human BECs: tetravalent glycodendrimer **16** outperformed the original compound **1**, inhibiting over 60% of yeast adhesion to BECs. In contrast, tetravalent counterpart **11**, built onto a RAFT cyclopeptide scaffold, showed only approximately half the activity (30% reduction in adhesion), being a less effective inhibitor than monomer **1** (with 45% reduction). Interestingly, an increase in the number of copies of glycomimetic **1** did not result in an improvement of anti-adhesion activity, with hexavalent **14** and hexadecavalent compounds **18** and **19** performing comparably or slightly worse than original inhibitor **1**, regardless of the chemical structure of the core scaffolds. As discussed earlier, remarkable increases in affinity toward isolated bacterial and fungal lectins have been reported upon multivalent display of carbohydrate epitopes. However, it is possible that whole bacterial or fungal cells, with the inherent complexity of the cell wall, present a more challenging environment for large glycoclusters to be able to access optimal three-dimensional presentations. A glycomimetic approach, guided by structural knowledge of the target proteins, may promote more effective interactions. In fact, the SEM images of *C. albicans* yeast cells treated with glycoclusters **14** and **16** showed distinctly different yeast adhesion patterns when compared to original compound **1**, even if these compounds were all effective inhibitors of yeast adhesion to BECs. Overall, this work highlights that increasing the number of glycomimetic epitopes presented by multivalent scaffolds does not necessarily result in an increase of biological activity. In particular, for the inhibitors of the adhesion of *C. albicans* discussed herein, lower valency dendrimeric presentations yielded the most promising results.

**Table 1:** Summary of Anti-Adhesion Activity of Lead Compound **1** Glycomimetic **1**, the Lower Valency Glycoclusters **11**, **14**, and **16**, and the Higher Valency Glycoclusters **18** and **19**

compound	number of copies of the glycomimetic	exclusion assay: % reduction in adhesion	competition assay: % reduction in adhesion
**1**	1	45	48
**11** (R_4_)	4	30	33
**14** (R_6_)	6	47	42
**16** (D_4_)	4	64	62
**18** (RR_16_)	16	33	38
**19** (DD_16_)	16	34	40

## Experimental Procedures

### Synthesis

#### General Methods

All reagents for synthesis were bought commercially and used without further purification. Reactions were monitored with thin layer chromatography (TLC) on Merck Silica Gel F_254_ plates. Detection was effected by UV (λ = 254 nm) or charring in a mixture of 5% sulfuric acid–ethanol. NMR spectra were recorded using Bruker Ascend 500 and Avance III spectrometers at 293 K. All chemical shifts were referenced relative to the relevant deuterated solvent residual peaks. Assignments of the NMR spectra were deduced using ^1^H NMR and ^13^C NMR, along with 2D experiments (COSY, HSQC, and HMBC). Chemical shifts are reported in ppm. Flash chromatography was performed with Merck Silica Gel 60. Microwave (μW) reactions were carried out using a CEM Discover Microwave Synthesizer. Optical rotations were obtained from an AA-100 polarimeter and [α]_D_ values are given in 10^–1^ cm^2^·g^–1^. High resolution mass spectrometry (HRMS) was performed on an Agilent-LC 1200 Series coupled to a 6210 Agilent Time-Of-Flight (TOF) and a Waters Xevo G2-S QToF mass spectrometers equipped with an electrospray source in both positive and negative (ESI±) modes. SEM images were taken on a HITACHI S-3200N Scanning Electron Microscope. FT-IR spectra were recorded on a PerkinElmer Spectrum 100 spectrophotometer, via ATR as a solid on a zinc selenide crystal or as a film on NaCl plates in the region 4000–400 cm^–1^. Spectroscopic data for all compounds are provided in the Supporting Information.

#### *N*,*N*′-Di(2,3,4,6-tetra-*O*-acetyl-β-d-galactopyranosyl-1,2,3-triazol-4-ylmethylamide)-*N*″-(2-bromoacetamido)-5-aminobenzene-1,3-dicarboxamide (**5**)

Compound **4**(42) (1.128 g, 1.13 mmol) was dissolved in dry DCM (20 mL). NEt_3_ (0.19 mL, 1.35 mmol) was added to this solution. Bromoacetyl bromide (0.12 mL, 1.35 mmol) was dissolved in dry DCM (5 mL) in a separate round-bottom flask. The first solution was added to the second dropwise via a cannula, and the resulting reaction mixture was allowed to stir for 16 h. The reaction mixture was washed with water (20 mL), HCl (1 N, 20 mL), sat. NaHCO_3_ solution (20 mL), followed by brine (20 mL). The organic phase was dried (MgSO_4_), and the solvent was removed *in vacuo* to obtain the pure product **5** without further purification as a brown, sticky solid (1.056 g, 83%). *R*_f_ = 0.65 (DCM, 5% MeOH). –4.0 (c 1.0, DCM). ^1^H NMR (500 MHz, CDCl_3_) δ 9.10 (s, 1H, N*H*COCH_2_Br), 8.09–7.90 (m, 6H, triaz-H, CON*H*CH_2_–triaz and Ar–H), 7.75 (s, 1H, Ar–H), 5.93 (d, *J* = 9.2 Hz, 2H, H-1), 5.60 (t, *J* = 9.7 Hz, 2H, H-2), 5.54 (d, *J* = 2.9 Hz, 2H, H-4), 5.32–5.26 (m, 2H, H-3), 4.67 (ddd, *J* = 39.3, 15.1, 5.4 Hz, 4H, CH_2_–triaz), 4.31 (t, *J* = 6.4 Hz, 2H, H-5), 4.22–4.11 (m, 4H, H-6 and H-6′), 3.98 (s, 2H, C*H*_*2*_-Br), 2.21 (s, 6H, OAc), 2.00 (app d, *J* = 2.7 Hz, 12H, OAc × 2), 1.82 (s, 6H, OAc). ^13^C NMR (125 MHz, CDCl_3_) δ 170.4 (CO of OAc), 170.1 (CO of OAc), 169.8 (CO of OAc), 169.4 (CO of OAc), 166.5 (*C*ONHCH_2_–triaz), 165.0 (*C*OCH_2_Br), 145.6 (*C*-triaz), 138.3 (Ar-*C*), 135.0 (Ar-*C*), 121.6 (*C*H-triaz), 121.4 (Ar-*C*H), 121.2 (Ar-*C*H), 86.2 (C-1), 74.0 (C-5), 70.8 (C-3), 68.1 (C-2), 66.8 (C-4), 61.2 (C-6), 35.5 (*C*H_2_-triaz), 29.6 (NHCO*C*H_2_Br), 20.7 (CH_3_ of OAc), 20.6 (CH_3_ of OAc), 20.5 (CH_3_ of OAc), 20.3 (CH_3_ of OAc). IR (film on NaCl): 3345, 3087, 2975, 1752, 1651, 1536, 1446, 1371, 1227, 1063, 924 732 cm^–1^. HRMS (ESI+): *m*/*z* calculated for C_44_H_52_BrN_12_O_21_ + H^+^ [M+H^+^]: 1122.2539, found 1122.2545.

#### *N*,*N*′-Di(2,3,4,6-tetra-*O*-acetyl-β-d-galactopyranosyl-1,2,3-triazol-4-ylmethyl amide)-*N*″-(2-azidoacetamido)-5-aminobenzene-1,3-dicarboxamide (**6**)

Compound **5** (231 mg, 0.206 mmol) and NaN_3_ (30 mg, 0.412 mmol) were dissolved in anhydrous DMF (10 mL) and heated to 80 °C. The reaction mixture was allowed to stir for 16 h. The solvent was removed *in vacuo*, and the resulting residue was redissolved in DCM (20 mL) and washed with brine (20 mL × 3). The organic phase was dried over MgSO_4_ and the solvent was removed *in vacuo* to obtain the pure product **6** without further purification as a yellow solid (1.056 g, 83%). *R*_f_ = 0.41 (DCM:MeOH 9:1). –5.6 (c 0.9, DCM). ^1^H NMR (500 MHz, CDCl_3_) δ 9.10 (s, 1H, N*H*COCH_2_N_3_), 8.18 (s, 2H, N*H*CH_2_CCH), 8.02 (s, 2H, Ar–H), 7.97 (s, 2H, C*H*-triaz), 7.82 (s, 1H, Ar–H), 5.95 (d, *J* = 9.2 Hz, 2H, H-1), 5.61 (t, *J* = 9.7 Hz, 2H, H-2), 5.56 (d, *J* = 3.1 Hz, 2H, H-4), 5.32 (dd, *J* = 10.1, 3.5 Hz, 2H, H-3), 4.67 (ddd, *J* = 20.4, 15.4, 5.5 Hz, 4H, C*H*_*2*_-triaz), 4.34 (t, *J* = 6.6 Hz, 2H, H-5), 4.23–4.13 (m, 4H, H-6 and H-6′), 4.06 (s, 2H, C*H*_*2*_-N_3_), 2.21 (s, 6H, OAc), 2.01 (s, 12H, OAc × 2), 1.82 (s, 6H, OAc). ^13^C NMR (125 MHz, CDCl_3_) δ 170.4 (CO of OAc), 170.1 (CO of OAc), 169.9 (CO of OAc), 169.3 (CO of OAc), 166.5 (*C*ONHCH_2_CCH), 166.3 (*C*OCH_2_N_3_), 145.5 (*C*-triaz), 138.0 (Ar-*C*), 134.9 (Ar-*C*), 121.6 (Ar-*C*H and *C*H-triaz), 121.4 (Ar-*C*H), 86.1 (C-1), 73.9 (C-5), 70.8 (C-3), 68.1 (C-2), 66.9 (C-4), 61.2 (C-6), 52.5 (*C*H_2_N_3_), 35.4 (*C*H_2_-triaz), 20.7 (CH_3_ of OAc), 20.6 (CH_3_ of OAc), 20.5 (CH_3_ of OAc), 20.2 (CH_3_ of OAc). IR (film on NaCl): 3342, 2942, 2110, 1747, 1655, 1528, 1427, 1368, 1211, 1046, 923, 733 cm^–1^. HRMS (ESI+): *m*/*z* calculated for C_44_H_52_N_12_O_21_ + Na^+^ [M+Na^+^]: 1107.3268, found 1107.3303.

#### Triethylene Glycol Dipropargyl Ether (**7**)

(Adapted from Feng et al.^45^) Triethylene glycol (1 mL, 7.33 mmol) was dissolved in dry THF under N_2_. NaH (60% dispersion in oil, 1.172 g, 29.3 mmol) was added, and the reaction mixture was allowed to stir for 1 h. Propargyl bromide (4 mL, 44 mmol) was added, and the reaction mixture was allowed to stir for at rt for 48 h. The solvent was removed *in vacuo*, and the resulting residue was dissolved in DCM (20 mL) and washed with water (10 mL × 2), dried (MgSO_4_), filtered, and concentrated *in vacuo*. The crude product was purified by silica gel column chromatography (EtOAc:Pet Ether 2:1) to give the pure product as a yellow oil (1.526 g, 92%). ^1^H NMR (500 MHz, CDCl_3_) δ 4.20 (d, *J* = 2.4 Hz, 4H, C*H*_*2*_CCH), 3.72–3.51 (m, 12H, OC*H*_*2*_ × 6), 2.42 (t, *J* = 2.4 Hz, 2H, CH_2_CC*H*). ^1^H NMR and ^13^C NMR spectroscopic data corresponded to that found in the literature.^23^

#### *N*,*N*′-Di(2,3,4,6-tetra-*O*-acetyl-β-d-galactopyranosyl-1,2,3-triazol-4-ylmethyl amide)-*N*″-(2-4-((2-(2-(2-(prop-2-yn-1-yloxy)ethoxy)ethoxy)ethoxy)methyl)-1*H*-1,2,3-triazol-1-yl)acetamido)-5-aminobenzene-1,3-dicarboxamide (**8**)

5 mL of a solution of compound **6** (prepared by dissolving 293 mg in 22 mL CH_3_CN and 5 mL of water, [1 mM]) was combined with 5 mL of a solution of compound **7** (prepared by dissolving 223 mg in 22 mL CH_3_CN and 5 mL of water, [36 mM]) in a microwave flask. To this solution, 0.5 mL of a solution of sodium ascorbate (prepared by dissolving 180 mg in 2 mL of water) was added, followed by 0.5 mL of a solution of copper sulfate pentahydrate (prepared by dissolving 70 mg in 2 mL of water). The mixture was allowed to react in the MW at 100 °C for 10 min. TLC was used to monitor the reaction; staining the TLC using potassium permanganate solution displayed the product as a bright yellow spot, whereas the starting compound **6** was a white spot and the diclick product was a brown spot on the TLC plate after staining. This procedure was repeated until all the stock solutions were used. The reaction mixture was evaporated and the crude product was purified by silicia gel column chromatography (DCM:MeOH 100:0-90:10) to give the product as a pale yellow solid (160 mg, 45%). *R*_f_ = 0.65 (DCM:MeOH 9:1). –1 (c 1, DCM). ^1^H NMR (500 MHz, CDCl_3_) δ 9.63 (s, 1H, N*H*CH_2_N_3_), 8.10 (bs, 2H, N*H*CH_2_–triaz), 7.91 (s, 2H, C*H*-triaz), 7.80 (s, 1H, C*H*-triaz), 7.78 (s, 2H, Ar–H), 7.73 (s, 1H, Ar–H), 5.85 (d, *J* = 9.2 Hz, 2H, H-1), 5.57 (t, *J* = 9.8 Hz, 2H, H-2), 5.47 (d, *J* = 2.7 Hz, 2H, H-4), 5.23–5.19 (m, 4H, H-3 and C*H*_2_), 4.65–4.56 (m, 6H, C*H*_*2*_-triaz), 4.24 (t, *J* = 6.3 Hz, 2H, H-5), 4.13–4.04 (m, 6H, H-6, H-6′ and OC*H*_2_CCH), 3.61–3.53 (m, 12H, 3 × OC*H*_2_C*H*_2_O), 2.32 (s, 1H, CC*H*), 2.12 (s, 6H, CH_3_ of OAc), 1.94 (s, 12H, CH_3_ of OAc), 1.73 (s, 6H, CH_3_ of OAc). ^13^C NMR (125 MHz, CDCl_3_) δ 169.3 (CO of OAc), 169.1 (CO of OAc), 168.9 (CO of OAc), 168.4 (CO of OAc), 165.6 (*C*ONHCH_2_–triaz), 163.3 (*C*OCH_2_–triaz), 144.5 (*C*-triaz), 143.9 (*C*-triaz), 137.1 (Ar-*C*), 133.9 (Ar-*C*), 124.2 (*CH*-triaz), 120.7 (*C*H-triaz and Ar-*C*H), 120.4 (Ar-*C*H), 85.1 (C-1), 78.5 (*C*CH), 73.9 (C-5), 72.8 (*C*CH), 69.7 (C-3), 69.4 (CH_2_ × 2), 69.2 (CH_2_), 68.8 (CH_2_), 68.0 (C-2), 67.0 (C-4), 65.9 (NHCO*C*H_2_N_3_), 60.1 (C-6), 57.3 (O*C*H_2_CCH), 52.4 (CH_2_), 34.5 (*C*H_2_-triaz), 19.6 (CH_3_ of OAc × 2), 19.5 (CH_3_ of OAc), 19.2 (CH_3_ of OAc). IR (film on NaCl): 3290, 3145, 2917, 2115, 1752, 1657, 1535, 1447, 1370, 1225, 1093, 1054, 924 cm^–1^. HRMS (ESI+): *m*/*z* calculated for C_56_H_70_N_12_O_25_ + Na^+^ [M+Na^+^]: 1333.4473, found 1333.4456.

#### Synthesis of *N,N*′-di(β-d-galactopyranosyl-1,2,3-triazol-4-ylmethylamide)-*N*″-(2-4-((2-(2-(2-(prop-2-yn-1-yloxy)ethoxy)ethoxy)ethoxy)methyl)-1*H*-1,2,3-triazol-1-yl) acetamido)-5-aminobenzene-1,3-dicarboxamide (**9**)

Compound **8** (150 mg, 0.114 mmol) was dissolved in methanol/H_2_O (4 mL, 2 mL). NEt_3_ (0.1 mL) was added, and the reaction mixture was allowed to stir at 45 °C for 6 h. The solution was cooled, Amberlite H^+^ was added, and the mixture was allowed to stir for 30 min. The solution was filtered, and the solvent was removed *in vacuo*. Excess NEt_3_ was removed using the Schlenk line. The product was freeze-dried overnight to yield the pure product **9** as a white solid (100 mg, 90%). +7 (c 1, MeOH:H_2_O 1:1). ^1^H NMR (500 MHz, D2O) δ 8.19 (s, 2H, C*H*-triaz), 8.02 (s, 1H, C*H*-triaz), 7.86 (s, 2H, Ar–H), 7.78 (s, 1H, Ar–H), 5.58 (d, *J* = 9.3 Hz, 2H, H-1), 5.34 (s, 2H, C*H*_2_), 4.58 (s, 2H, C*H*_*2*_-triaz), 4.58 (s, 2H, C*H*_*2*_-triaz), 4.55 (s, 4H, C*H*_*2*_-triaz), 4.13 (t, *J* = 9.8 Hz, 2H, H-2), 4.02 (s, 2H, OC*H*_2_CCH), 3.99 (s, 2H, H-4), 3.89 (m, 2H, H-5), 3.79–3.77 (m, 2H, H-3), 3.68–3.63 (m. 4H, H-6 and H-6′), 3.61 (bs, 2H, OC*H*_2_), 3.57–3.53 (m, 10H, 3 × C*H*_2_), 2.67 (s, 1H, CC*H*). ^13^C NMR (125 MHz, CDCl_3_) δ 168.4 (*C*ONHCH_2_–triaz), 166.1 (*C*OCH_2_–triaz), 144.9 (*C*-triaz), 144.2 (*C*-triaz), 137.6 (Ar-*C*), 134.6 (Ar-*C*), 126.6 (*CH*-triaz), 123.1 (*C*H-triaz), 123.4 (Ar-*C*H × 2), 88.1 (C-1), 78.3 (*C*-5), 73.1 (C-3), 69.8 (C-2), 69.5 (CH_2_), 69.3 (CH_2_), 68.6 (C-4), 68.6, 68.5 (2 × CH_2_), 63.1 (NHCO*C*H_2_N_3_), 60.1 (C-6), 57.8 (O*C*H_2_CCH), 52.5 (CH_2_), 34.1 (*C*H_2_-triaz). IR (ATR): 3269, 2927, 2491, 1704, 1645, 1598, 1538, 1447, 1347, 1227, 1090, 1052, 890 cm^–1^. HRMS (ESI+): *m*/*z* calculated for C_40_H_54_N_12_O_17_ + Na^+^ [M+Na^+^]: 997.3628, found 997.3615.

### General Procedure for CuAAC Ligation for Glycoclusters

A solution of CuSO_4_·5H_2_O, tris(3-hydroxypropyltriazolylmethyl)amine (THPTA) and sodium ascorbate in phosphate-buffered saline (PBS, pH 7.5, 10 mM) was added to a solution of the azido-containing compound and the alkyne-containing compound (1.5 equiv per azide) in DMF at room temperature. The mixture was degassed under argon and stirred at room temperature for 1 h under argon. UPLC analysis was used to determine the end point of the reaction. Chelex resin was added to the reaction mixture, which was stirred for an additional 30 min to remove excess Cu^2+^ ions. The reaction mixture was then purified by semipreparative RP-HPLC to afford pure compounds as white fluffy solids after lyophilization.

#### Compound **11**

A solution of CuSO_4_·5H_2_O (0.79 mg, 3.2 μmol), THPTA (2.8 mg, 6.4 μmol), and sodium ascorbate (3.8 mg, 19.2 μmol) in PBS buffer (400 μL, pH 7.5) was added to a solution of compound **10** (7.2 mg, 6.4 μmol) and **9** (2.74 mL of 10 mg/mL solution in PBS, 28.2 μmol) in 500 μL of DMF. The mixture was degassed under argon and stirred at room temperature for 1 h. UPLC analysis showed the reaction was not complete. A further 2 equiv of **9** was added (1.24 mL of 10 mg/mL solution in PBS, 13.0 μmol). The mixture was degassed under argon and stirred at room temperature for 1 h. UPLC analysis showed complete coupling. Chelex resin was added to the reaction mixture, which was stirred for an additional 30 min and purified by semipreparative RP-HPLC (5–40% CH_3_CN in 15 min) to afford the desired compound **11** as a white fluffy solid after lyophilization (21 mg, 65%). ^1^H NMR (500 MHz, D_2_O) δ 8.47 (s, 1H), 8.22 (s, 8H), 8.05 (s, 4H), 7.95–7.85 (m, 8H), 7.87–7.71 (m, 8H), 5.64 (d, *J* = 9.2 Hz, 8H), 5.37 (s, 8H), 4.59 (s, 26H), 4.50 (s, 9H), 4.44–4.31 (m, 5H), 4.31–4.18 (m, 19H), 4.08 (d, *J* = 3.3 Hz, 8H), 4.07–4.00 (m, 2H), 3.97 (t, *J* = 6.1 Hz, 8H), 3.86 (dd, *J* = 9.8, 3.3 Hz, 10H), 3.75 (d, *J* = 6.0 Hz, 21H), 3.67–3.48 (m, 53H), 2.96 (t, *J* = 7.6 Hz, 2H), 2.25 (s, 3H), 2.09–1.47 (m, 29H), 1.43–1.11 (m, 15H). HRMS (ESI+): *m*/*z* calculated for C_207_H_297_N_71_O_78_ + 4H^+^ [M+4H]^4+^: 1256.28586, found 1256.28652.

#### Compound **12**

Compound **11** (15.8 mg, 3.15 μmol) and *N*-succinimidyl pentynoate (0.9 mg, 4.6 μmol) were dissolved in dry DMF (1 mL). Diisopropylethylamine (2 μL × 3, mmol) were added until the solution was at pH 9. The mixture was stirred at room temperature for 1 h after which UPLC analysis showed complete conversion. H_2_O (3 mL) was added to the mixture, which was then purified by semipreparative RP-HPLC (5–40% CH_3_CN in 15 min) to afford the desired compound **12** as a white fluffy solid after lyophilization (15.5 mg, 97%).

#### Compound **14**

A solution of CuSO_4_·5H_2_O (0.6 mg, 2.4 μmol), THPTA (2.1 mg, 4.8 μmol), and sodium ascorbate (2.9 mg, 28.8 μmol) in PBS buffer (400 μL, pH 7.5) was added to a solution of the **13** (4 mg, 2.4 μmol) and **9** (21 mg, 21.6 μmol) in 500 μL of DMF. The mixture was degassed under argon and stirred at room temperature for 1 h. UPLC analysis showed complete coupling. Chelex resin was added to the reaction mixture, which was stirred for an additional 30 min and purified by semipreparative RP-HPLC (5–40% CH_3_CN in 15 min) to afford the desired compound **4.55** as a white fluffy solid after lyophilization (6.2 mg, 34%). ^1^H NMR (500 MHz, DMSO) δ = 10.71 (s, 9H), 9.02 (m, 14H), 8.31–8.11 (m, 17H), 8.01 (m, 17H), 7.95–7.65 (m, 26H), 5.37 (d, *J* = 9.2, 12H, H-1), 5.26 (d, *J* = 8.1, 14H), 5.13 (d, *J* = 5.8, 16H), 4.94 (m, 20H), 4.60 (t, *J* = 5.6, 13H), 4.55 (d, *J* = 5.5, 9H), 4.45 (dd, *J* = 12.0, 7.6, 29H), 4.16 (bs, 26H), 3.91 (dd, *J* = 15.1, 9.2, 18H), 3.65 (m, 11H), 3.60 (m, 12H), 3.41 (m, 55H), 2.97 (bs, 20H), 2.58–2.53 (m, 7H), 2.37–2.25 (m, 6H), 2.1–1.5 (m, 54H), 1.5–1.0 (m, 65H), 0.81–0.73 (m, 9H). MALDI-TOF-MS [M + H]^+^: *m*/*z* calculated for C_314_H_442_N_110_O_122_ + H^+^: 7706.184, found 7706.360.

#### Compound **16**

A solution of CuSO_4_·5H_2_O (0.79 mg, 3.2 μmol), THPTA (2.8 mg, 6.4 μmol), and sodium ascorbate (3.8 mg, 19.2 μmol) in PBS buffer (400 μL, pH 7.5) was added to a solution of the **15** (7 mg, 6.8 μmol) and **9** (3.9 mL of 10 mg/mL solution in PBS, 40.7 μmol) in 500 μL of DMF. The mixture was degassed under argon and stirred at room temperature for 1 h. UPLC analysis showed complete coupling. Chelex resin was added to the reaction mixture, which was stirred for an additional 30 min and purified by semipreparative RP-HPLC (5–40% CH_3_CN in 15 min) to afford the desired compound **4.57** as a white fluffy solid after lyophilization (25 mg, 78%). ^1^H NMR (500 MHz, D_2_O) δ 8.47 (s, 1H), 8.21 (s, 8H), 8.05 (d, *J* = 2.4 Hz, 4H), 7.97–7.88 (m, 10H), 7.85–7.78 (m, 6H), 5.65 (d, *J* = 9.1 Hz, 8H), 5.38 (s, 8H), 5.22 (d, *J* = 8.2 Hz, 4H), 4.68–4.55 (m, 24H), 4.52 (d, *J* = 5.2 Hz, 9H), 4.30–4.14 (m, 17H), 4.08 (d, *J* = 3.3 Hz, 8H), 3.97 (t, *J* = 6.1 Hz, 8H), 3.86 (dd, *J* = 9.8, 3.3 Hz, 9H), 3.75 (d, *J* = 6.0 Hz, 17H), 3.67–3.47 (m, 51H), 3.00 (s, 2H), 2.94 (t, *J* = 7.6 Hz, 2H), 1.88–1.52 (m, 15H), 1.48–1.06 (m, 14H). HRMS (ESI+): *m*/*z* calculated for C_191_H_274_N_68_O_75_ + 4H^+^ [M+4H]^4+^: 1179.99237, found 1179.99202.

#### Compound **17**

Compound **16** (23.6 mg, 5.0 μmol) and *N*-succinimidyl pentynoate (1.46 mg, 7.5 μmol) were dissolved in dry DMF (1 mL). Diisopropylethylamine (2 μL × 3, mmol) were added until the solution was pH 9. The mixture was stirred at room temperature for 1 h after which UPLC analysis showed complete conversion. H_2_O (3 mL) was added to the mixture, which was then purified by semipreparative RP-HPLC (5–40% CH_3_CN in 15 min) to afford the compound **17** as a white fluffy solid after lyophilization (19.7 mg, 82%).

#### Compound **18**

A solution of CuSO_4_·5H_2_O (0.08 mg, 0.32 μmol), THPTA (0.27 mg, 0.62 μmol), and sodium ascorbate (0.37 mg, 1.9 μmol) in PBS buffer (400 μL, pH 7.5) was added to a solution of the **10** (0.7 mg, 0.62 μmol) and **12** (14 mg, 2.74 mmol) in 500 μL of DMF. The mixture was degassed under argon and stirred at room temperature for 1 h. UPLC analysis showed complete coupling. Chelex resin was added to the reaction mixture, which was stirred for an additional 30 min and purified by semipreparative RP-HPLC (5–40% CH_3_CN in 15 min) to afford the desired compound **18** as a white fluffy solid after lyophilization (12 mg, 89%). ^1^H NMR (500 MHz, DMSO) δ = 10.81 (s, 31H), 9.11 (s, 42H), 8.43 (s, 36H), 8.22 (s, 39H), 8.08 (m, 84H), 7.82 (m, 32H), 7.39 (s, 40H), 6.86 (s, 12H), 6.66 (m, 12H), 5.46 (d, *J* = 9.1, 32H, H-1), 5.36 (s, 34H), 5.23 (s, 34H), 5.02 (s, 36H), 4.67 (m, 70H), 4.53 (m, 123H), 4.27 (s, 64H), 4.11 (s, 27H), 4.00 (d, *J* = 4.2, 40H), 3.75 (s, 42H), 3.69 (t, *J* = 5.9, 40H), 3.52 (m, 266H), 3.00 (s, 29H), 2.80 (s, 24H), 2.64 (s, 17H), 2.40 (m, 31H), 2.07 (m, 66H), 1.73 (m, 125H), 1.50 (m, 40H), 1.23 (m, 156H), 0.86 (m, 17H). MALDI-TOF-MS [M + H]^+^: *m*/*z* calculated for C_895_H_1263_N_307_O_326_ + H^+^: 21539.474, found 21539.616.

#### Compound **19**

A solution of CuSO_4_·5H_2_O (0.1 mg, 0.46 μmol), THPTA (0.41 mg, 0.93 μmol), and sodium ascorbate (0.56 mg, 2.79 μmol) in PBS buffer (400 μL, pH 7.5) was added to a solution of the **15** (0.76 mg, 0.93 μmol) and **17** (19.7 mg, 4.11 mmol) in 500 μL of DMF. The mixture was degassed under argon and stirred at room temperature for 1 h. UPLC analysis showed complete coupling. Chelex resin was added to the reaction mixture, which was stirred for an additional 30 min and purified by semipreparative RP-HPLC (5–40% CH_3_CN in 15 min) to afford the desired compound **19** as a white fluffy solid after lyophilization (16.2 mg, 87%). ^1^H NMR (500 MHz, DMSO) δ = 10.86 (s, 24H), 9.17 (s, 32H), 8.59 (m, 26H), 8.28 (s, 34H), 8.13 (m, 80H), 7.87 (m, 28H), 7.34 (s, 10H), 7.07 (s, 10H), 5.52 (d, *J* = 9.1, 32H, H-1), 5.42 (s, 32H), 5.29 (s, 30H), 5.20 (s, 22H), 5.07 (s, 30H), 4.73 (m, 60H), 4.57 (m, 110H), 4.25 (m, 96H), 3.80 (s, 32H), 3.75 (t, *J* = 5.9, 30H), 3.57 (m, 260H), 3.23 (s, 16H), 3.06 (s, 24H), 2.92 (m, 18H), 2.71 (m, 15H), 2.43 (m, 18H), 1.79 (m, 80H), 1.30 (m, 82H), 0.91 (m, 6H). MALDI-TOF-MS [M + H]^+^: *m*/*z* calculated for C_815_H_1151_N_292_O_311_ + H^+^: 20015.637, found 20015.456.

### Biology

#### Fungal Strain

*C. albicans* (MEN, serotype B, clinical isolate from a corneal infection) was maintained on sabouraud dextrose agar, and cultures were grow to the stationary phase ((1–2) × 10^8^ cells/mL) overnight in YEPD broth (1% (w/v) yeast extract, 2% (w/v) bacteriological peptone, 2% (w/v) glucose) at 30 °C, and 200 rpm. Stationary phase yeast cells were harvested, washed with PBS, and resuspended at a density of 1 × 10^8^ cells/mL in PBS.

#### Buccal Epithelial Cells

Buccal epithelial cells (BECs) were harvested from healthy volunteers by gently scraping the inside of the cheek with a sterile tongue depressor. Cells were washed in PBS and resuspended at a density of 5 × 10^5^ cells/mL.

#### Toxicity

The compounds were incubated with *C. albicans* for 24 h; a dilution was performed (1/50) and 100 μL of the cell suspension was spread on YEPD agar plates. The compounds did not have a fungicidal effect on the yeast (Figure SI-1).

#### Adherence Assays

Yeast cells were mixed with 50:1 (yeast:BEC) in a final volume of 2 mL and incubated at 30 °C and 200 rpm for 90 min. The BEC/yeast cell mixture was harvested by passing through a polycarbonate membrane containing 30 μm pores which trapped the BECs but allowed unattached yeast cells to pass through. This was washed 2× with 10 mL PBS, and cells remaining on the membrane were collected and placed on glass slides which were left to air-dry overnight. The cells were heat-fixed and stained using 0.5% (w/v) crystal violet, rinsed using cold water to remove any surplus stain, and left to air-dry for 30 min. The number of *C. albicans* cells adhering to a sample of 200 BECs per treatment was assessed microscopically. In the exclusion assay, the yeast cells were incubated for 90 min in the presence of each compound at the given concentration. After this time, the yeast cells were harvested and washed twice with PBS before being resuspended in 1 mL PBS before being mixed with BECs (as described). In the competition assay format yeast cells, BECs and compound (at the given concentration) were coincubated for 90 min prior to harvesting.

#### SEM Sample Preparation

First, the yeast cells were incubated separately with compounds **1**, **14**, and **16**, along with a control of only yeast in PBS, for 24 h at 30 °C and 200 rpm. Sample preparation for SEM was then adapted from Manefield et al.^51^ The cells were fixed to a microscopic cover clip, with 2.5% (v/w) glutaraldehyde and kept at 4 °C for 12 h. The cells were then washed with sterile PBS (2×) dehydrated by sequential washing with ethanol series (70%, 95%, and 100%). The samples were then treated with hexamethyldisilazane (Sigma-Aldrich) and air-dried overnight in a desiccator. The cells were then sputtered with gold (6–12 nm) prior SEM imaging.

#### Biofilm

*C. albicans* cells were washed twice with PBS and enumerated before being resuspended in RPMI to give a cell density of (2 × 10^7^ cells/mL). Compounds were resuspended in PBS to give a concentration of 2 mg/mL. The compounds (250 μL) were mixed with RPMI (250 μL) in a 1:1 ratio to give a concentration of 1 mg/mL. PBS (250 μL) was mixed with RPMI (250 μL).

*C. albicans* cells (500 μL, 2 × 10^7^ cells/mL) were incubated with compounds (1 mg/mL) in a 1:1 ratio to give a final cell density to concentration ratio of 5 × 10^6^ cells/mL. The suspension was incubated for 3 h at rt and mixed by vortex every 20 min to prevent cells from falling out of solution. Cell suspension (100 μL) was added to wells of a 96-well flat bottom plate. The plate was incubated at 37 °C for 24 h. After incubation, the medium was removed from the wells with a pipet, with care taken not to disrupt the biofilm lining the bottom of the plate. Wells were washed twice with 100 μL aliquots of PBS to remove unbound yeast cells. Crystal violet (5% v/v) (100 μL) was added to each well and incubated at room temperature for 15 min. The crystal violet was removed by pipet as described before. Wells were washed twice with PBS (100 μL). The plates were incubated at room temperature for 2 h, with the lids left off and allowed to dry. Acetic acid (30% v/v) (100 μL) was added to each well. The plate was incubated for 10 min and a pipet was used to mix the contents of each well to ensure the biofilm/crystal violet was fully dissolved in the acetic acid. The plate was read at 550 nm in a 96-well plate reader. The percentage reduction in biofilm was calculated by dividing the absorbance value of the treated cells into that of the control.

#### Statistics

All experiments were performed on three independent occasions. In each assay the number of yeast cells adhering to 200 randomly chosen BECs was determined. Results are given as mean ± SEM (Standard Error of the Mean).

## Supporting Information Available

Structures and spectroscopic data; images and molecular weights (PDF)
