# Supplementary material for: Multivalent Presentations of Glycomimetic Inhibitor of the Adhesion of Fungal Pathogen Candida albicans to Human Buccal Epithelial Cells
Source: Bioconjug Chem. 2021 Apr 22;32(5):971–82. doi: 10.1021/acs.bioconjchem.1c00115 (PMC8154258; doi:10.1021/acs.bioconjchem.1c00115)
Supplement: Supplementary file 1 — bc1c00115_si_001.pdf [file bc1c00115_si_001.pdf]

Supporting Information

Multivalent Presentations of Glycomimetic Inhibitor of the Adhesion of Fungal Pathogen *Candida albicans* to Human Buccal Epithelial Cells

Harlei Martin[1], David Goyard[2], Anatte Margalit[3], Kyle Doherty[1], Olivier Renaudet[2], Kevin Kavanagh[3,4], and Trinidad Velasco-Torrijos[1,4].

[1]Department of Chemistry, Maynooth University, Maynooth, W23VP22, Co. Kildare, Ireland.

[2]DCM, UMR 5250, Université Grenoble Alpes, CNRS, 38000 Grenoble, France.

[3]Department of Biology, Maynooth University, Maynooth, W23VP22, Co. Kildare, Ireland.

[4]The Kathleen Lonsdale Institute for Human Health Research, Maynooth University, Maynooth, W23VP22, Co. Kildare, Ireland.

Corresponding author e-mail: trinidad.velascotorrijos@mu.ie

Contents:

Compound 5. ^{1}H NMR, ^{13}C NMR S1

Compound 6. ^{1}H NMR, ^{13}C NMR S2

Compound 8. ^{1}H NMR, ^{13}C NMR S3

Compound 9. ^{1}H NMR, ^{13}C NMR S4

Compound 11. ^{1}H NMR, RP-HPLC, HRMS (ESI^{+}-TOF) S5

Compound 14. ^{1}H NMR, RP-HPLC, MALDI-TOF S7

Compound 16. ^{1}H NMR, RP-HPLC, HRMS (ESI^{+}-TOF) S8

Compound 18. ^{1}H NMR, RP-HPLC, MALDI-TOF S10

Compound 19. ^{1}H NMR, RP-HPLC, MALDI-TOF S11

Table 1: Concentrations of compounds used in Adhesion assays S13

Images of *C. albicans* in the presence of **1** and **16** S13

Compound 5:

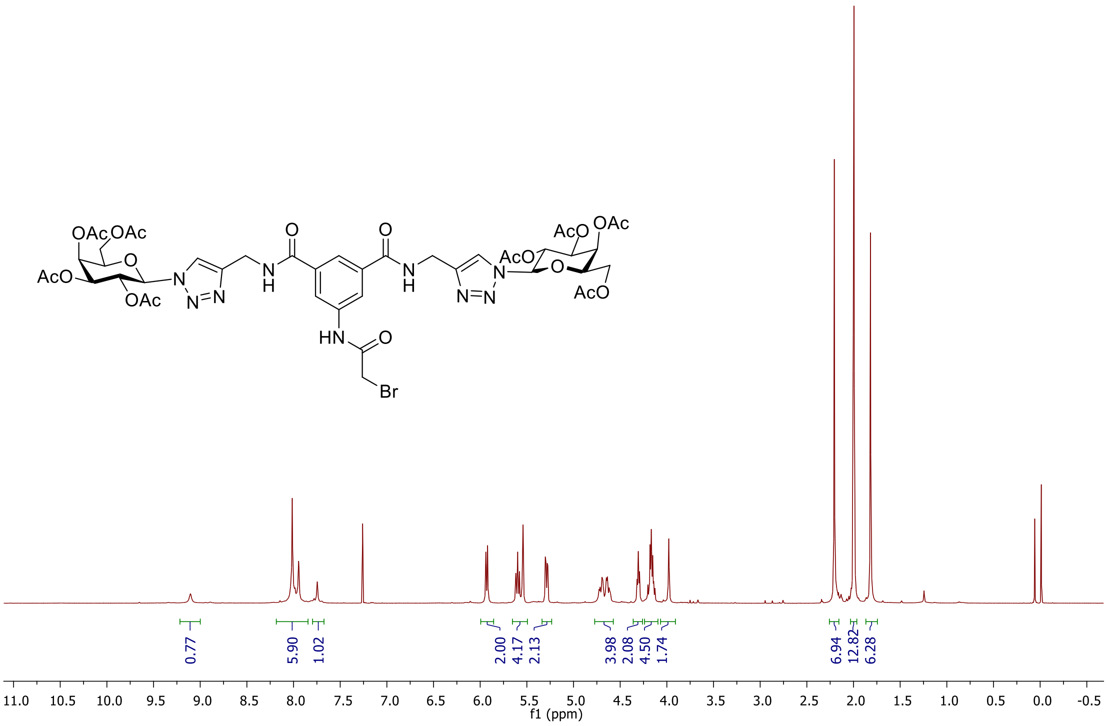

Figure S1: ^{1}H NMR spectra of compound **5** ($CDCl_3$, 500 MHz, 298 K).

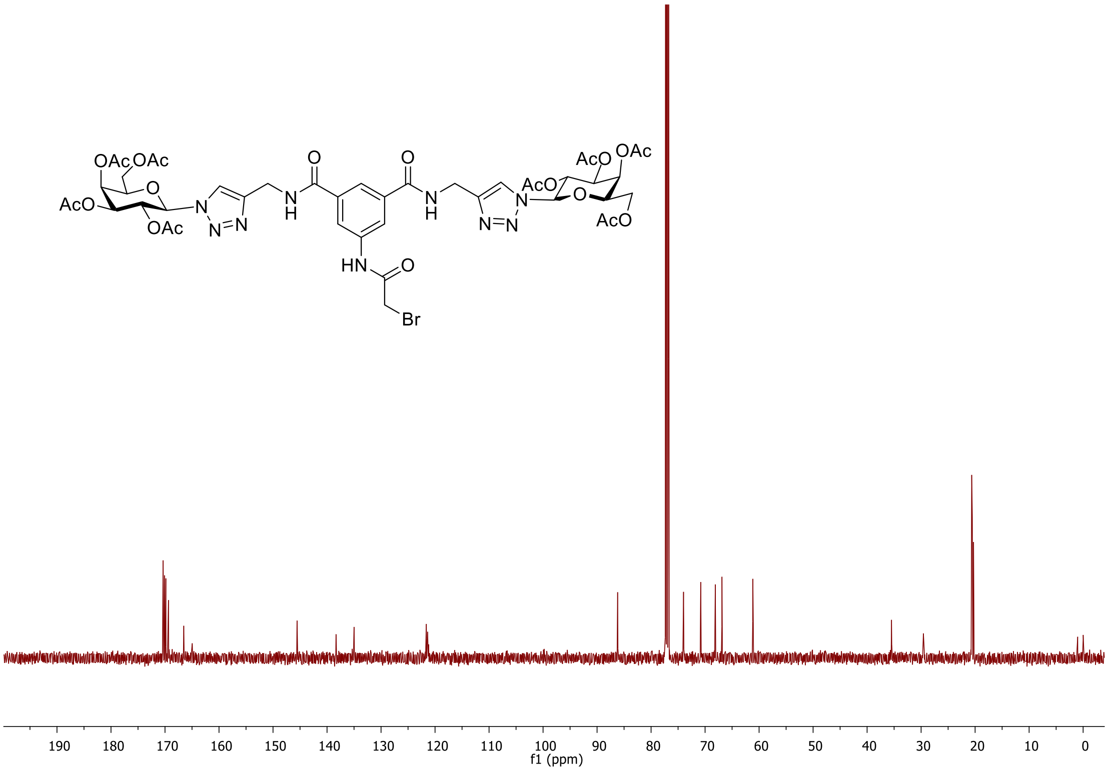

Figure S2: ^{13}C NMR spectra of compound **5** ($CDCl_3$, 500 MHz, 298 K).

Compound 6

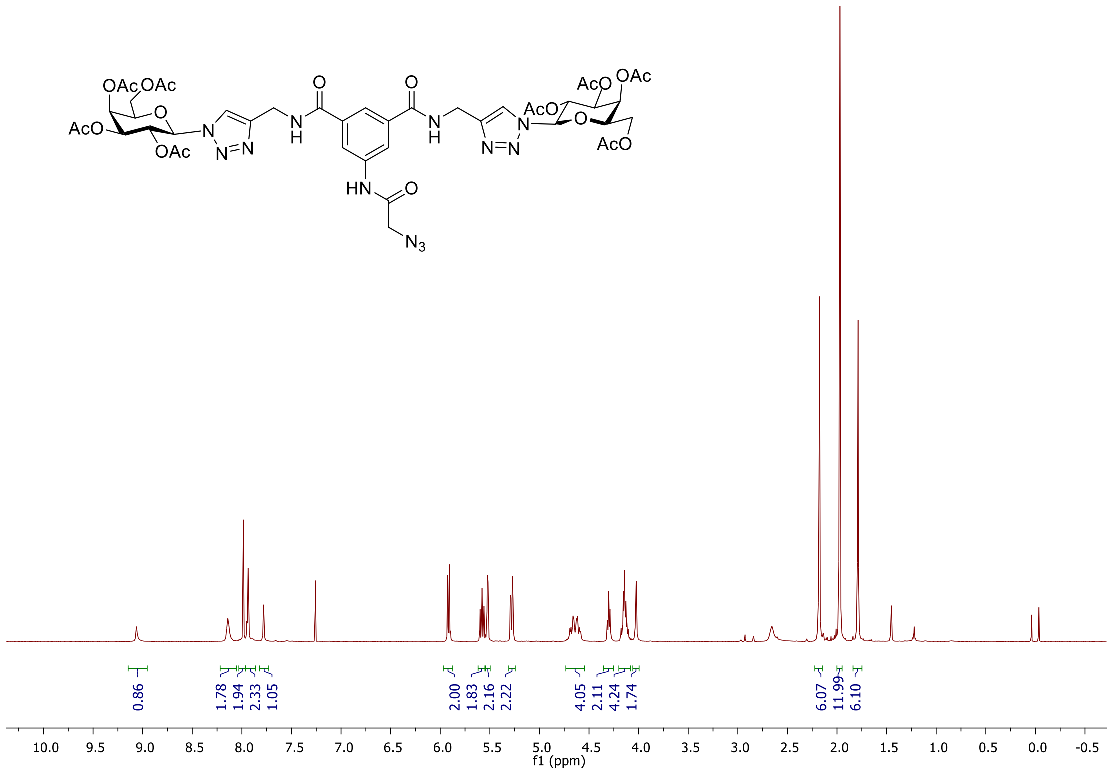

Figure S3: ^{1}H NMR spectra of compound **5** ($CDCl_3$, 500 MHz, 298 K).

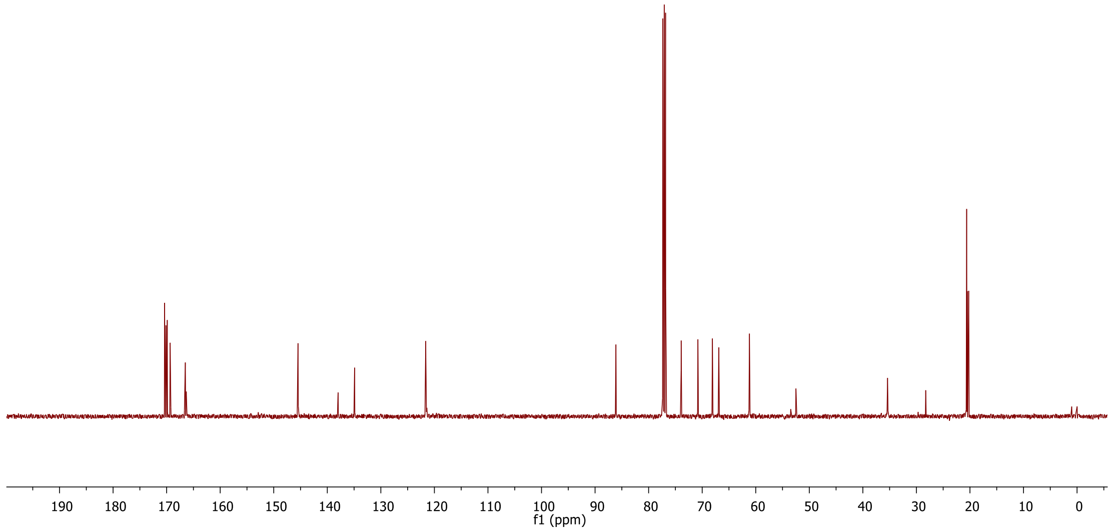

Figure S4: ^{13}C NMR spectra of compound **6** ($CDCl_3$, 500 MHz, 298 K).

Compound 8

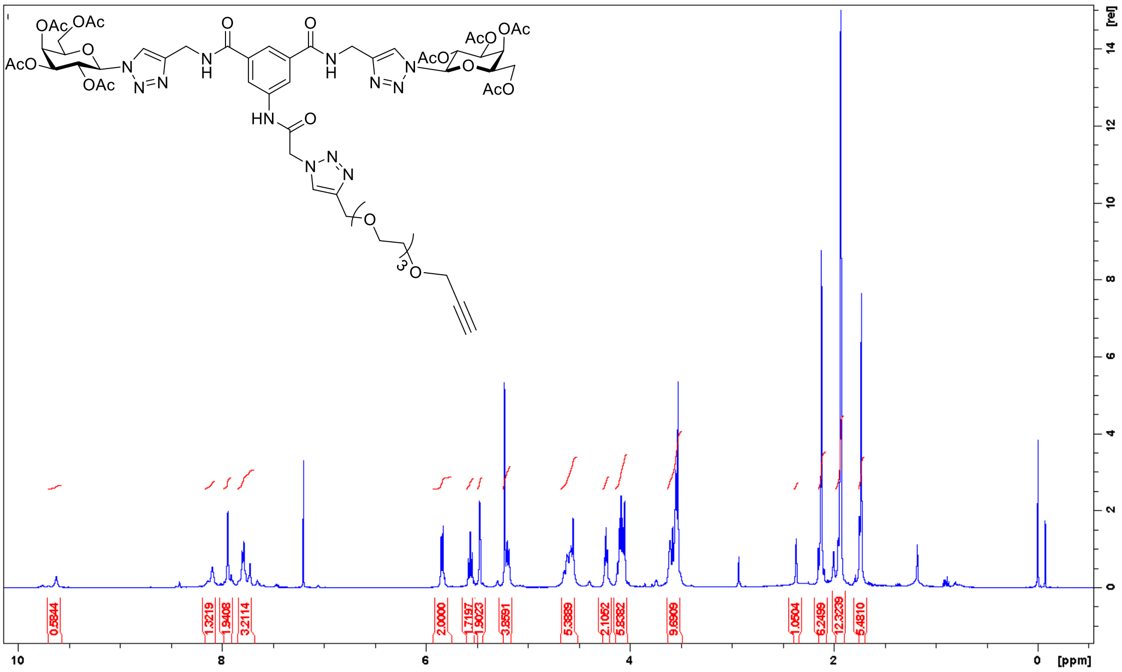

Figure S5: ^{1}H NMR spectra of compound **8** ($CDCl_3$, 500 MHz, 298 K).

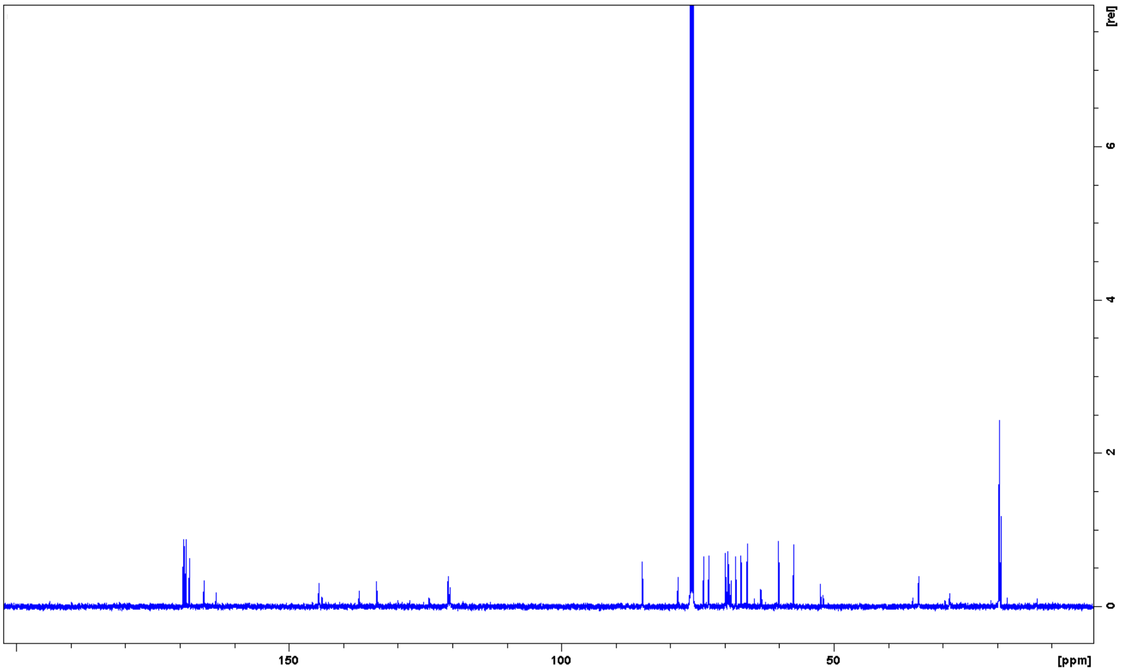

Figure S6: ^{13}C NMR spectra of compound **8** ($CDCl_3$, 500 MHz, 298 K).

Compound 9

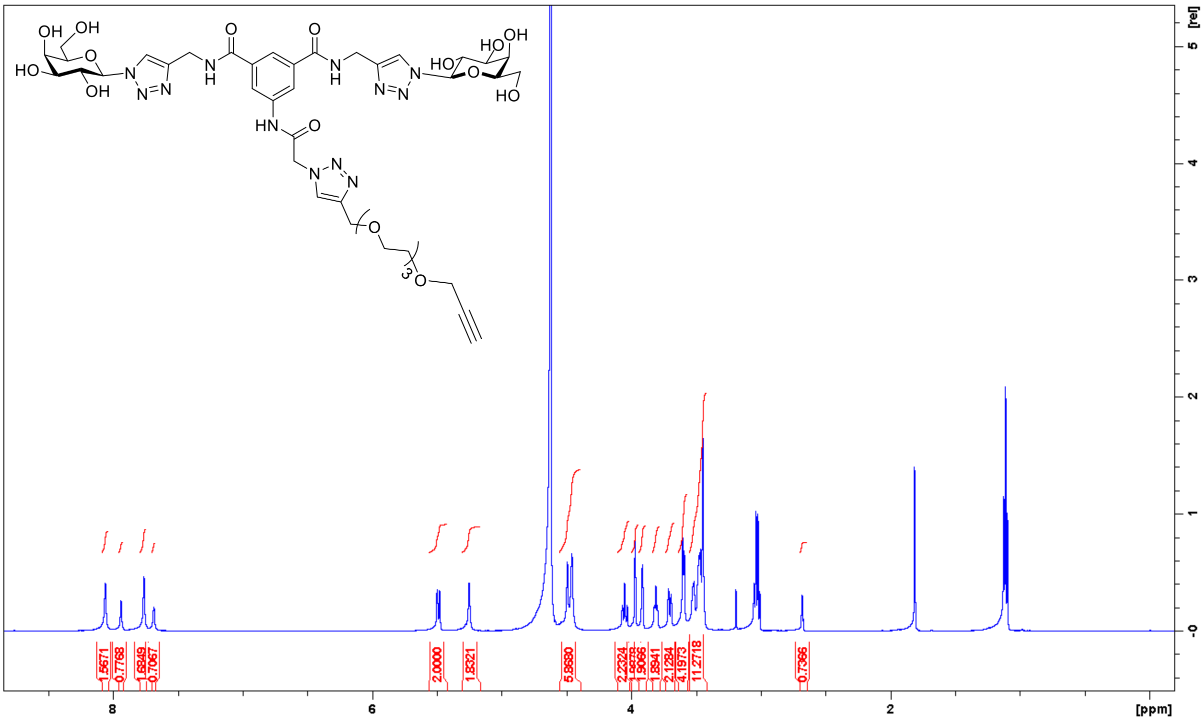

Figure S7: ^{1}H NMR spectra of compound **9** ($CDCl_3$, 500 MHz, 298 K).

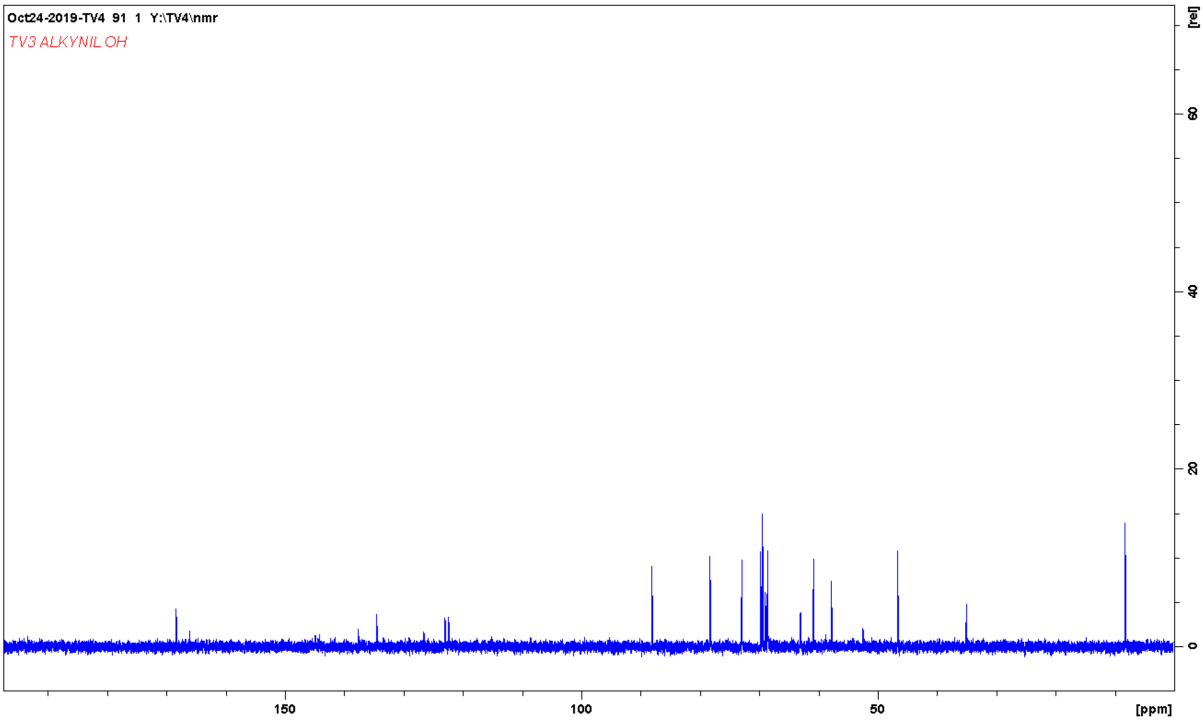

Figure S8: ^{13}C NMR spectra of compound **9** ($CDCl_3$, 500 MHz, 298 K).

For all following compounds:

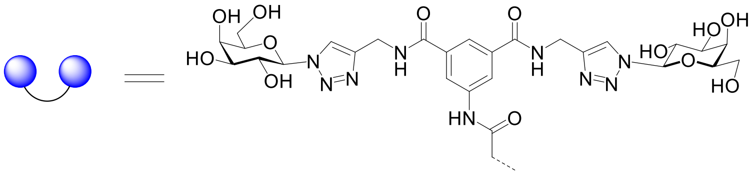

Compound 11

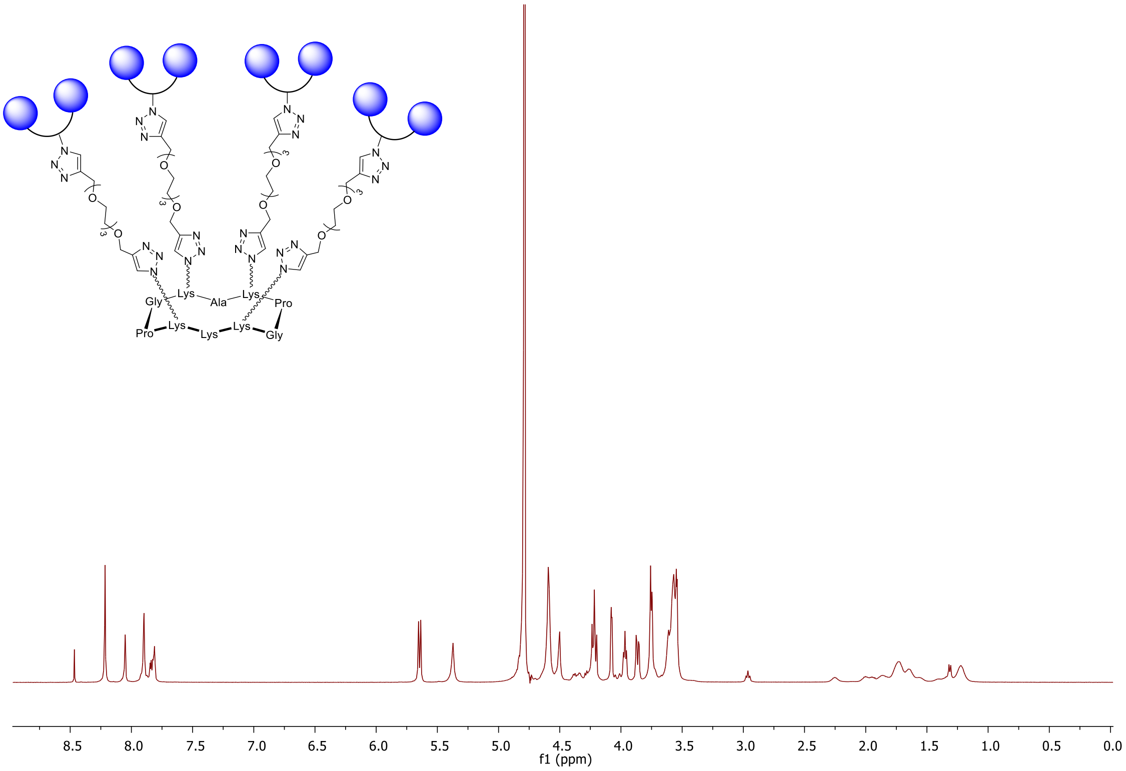

Figure S9: ¹H NMR spectra of compound **11** (D_2O, 500 MHz, 298 K).

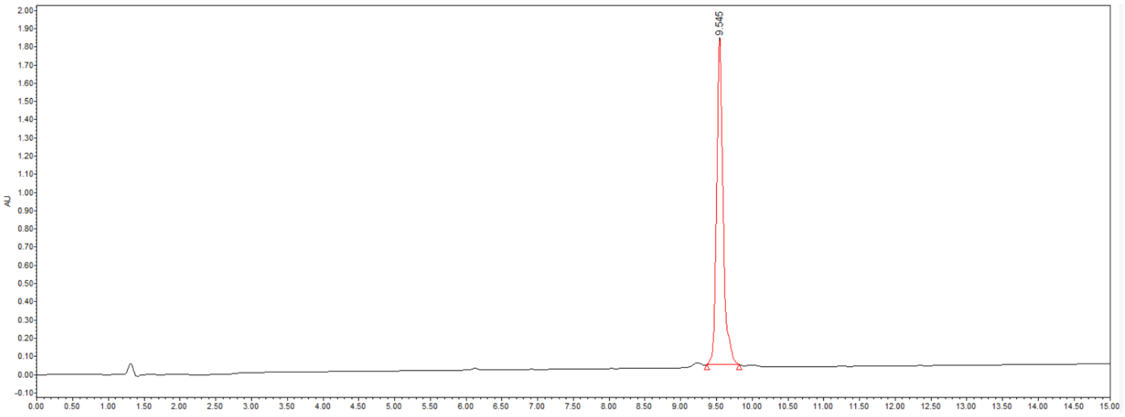

Figure S10: RP-HPLC of compound **11**.

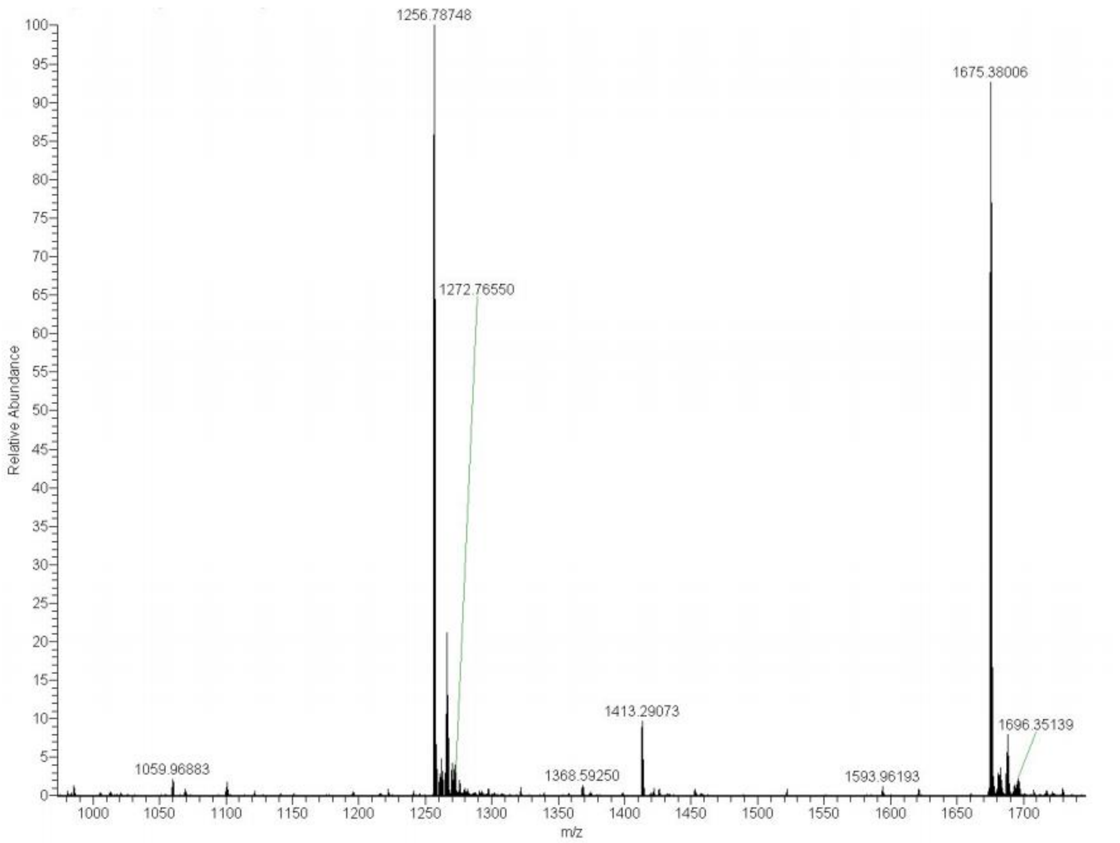

Figure S11: HRMS (ESI$^+$-TOF) of compound **11**.

Compound 14

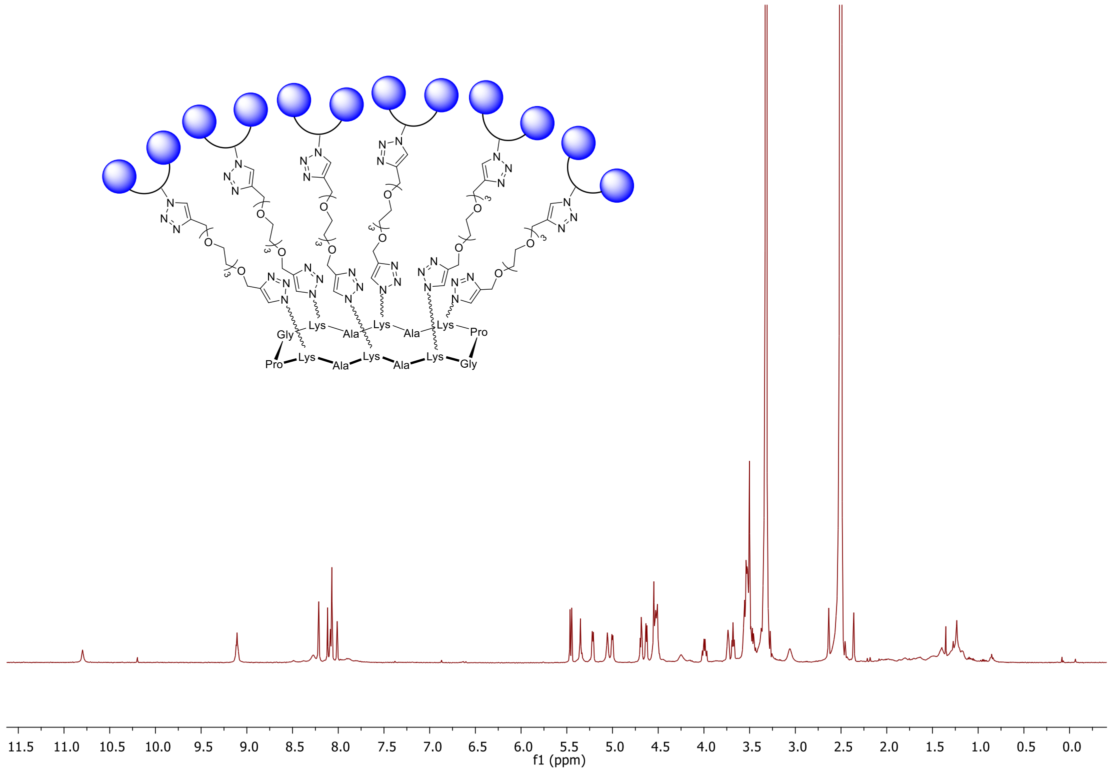

Figure S12: ^{1}H NMR spectra of compound **14** (DMSO, 500 MHz, 298 K).

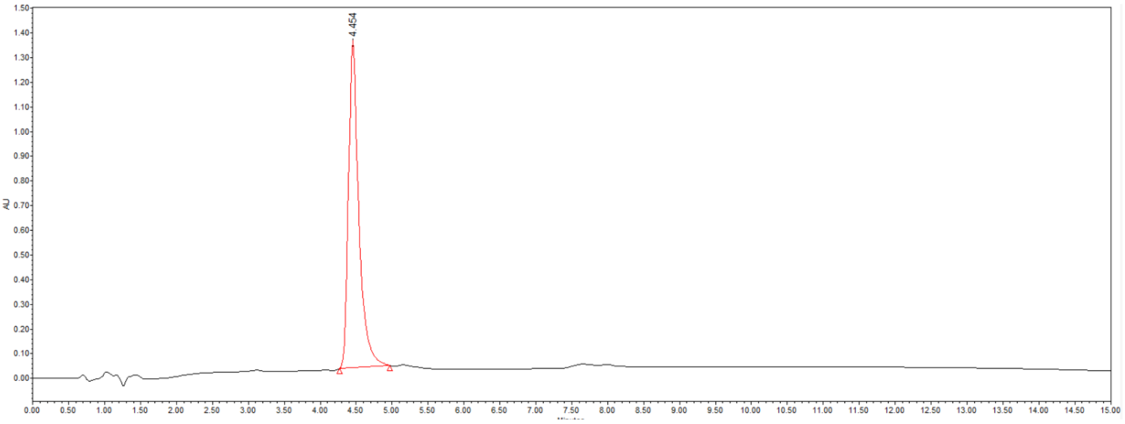

Figure S13: RP-HPLC of compound **14**.

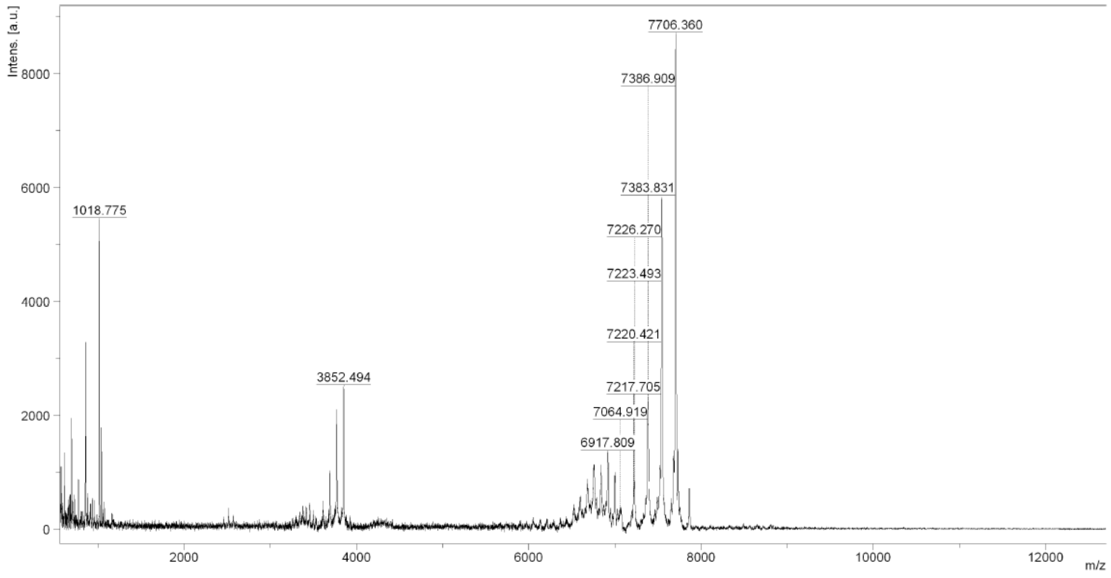

Figure S14: MALDI-TOF of compound **14**.

Compound 16

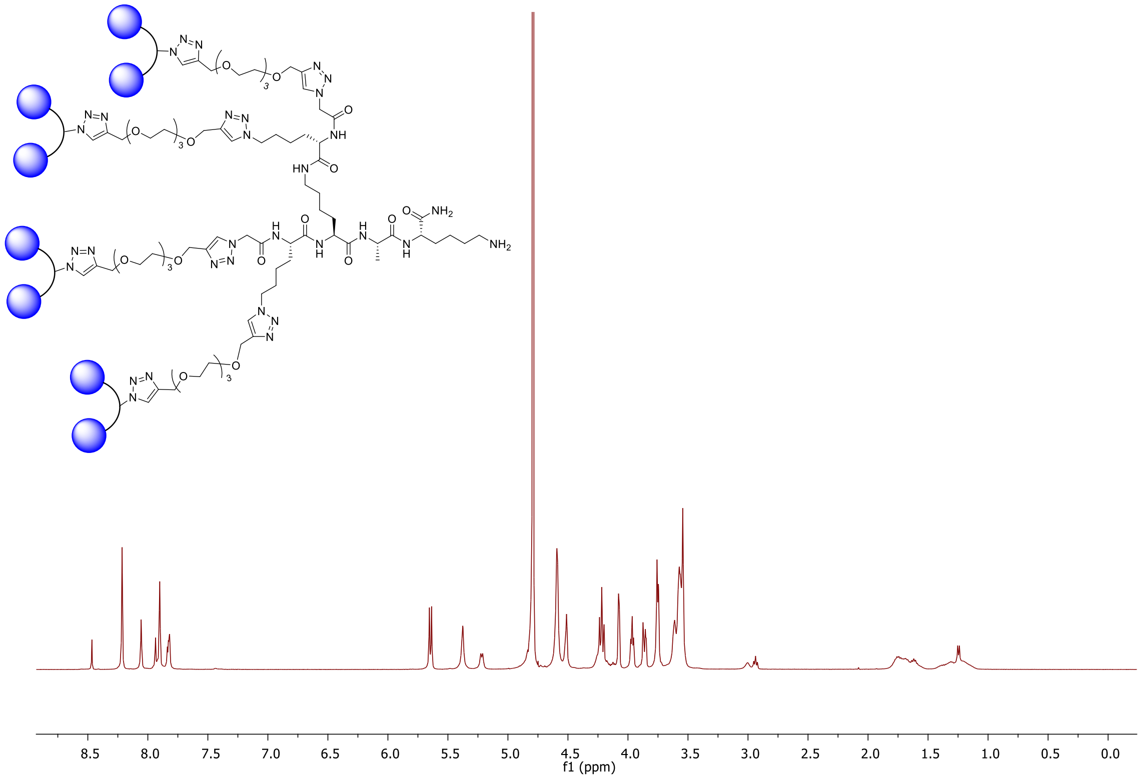

Figure S15: ^{1}H NMR spectra of compound **16** (D_2O, 500 MHz, 298 K).

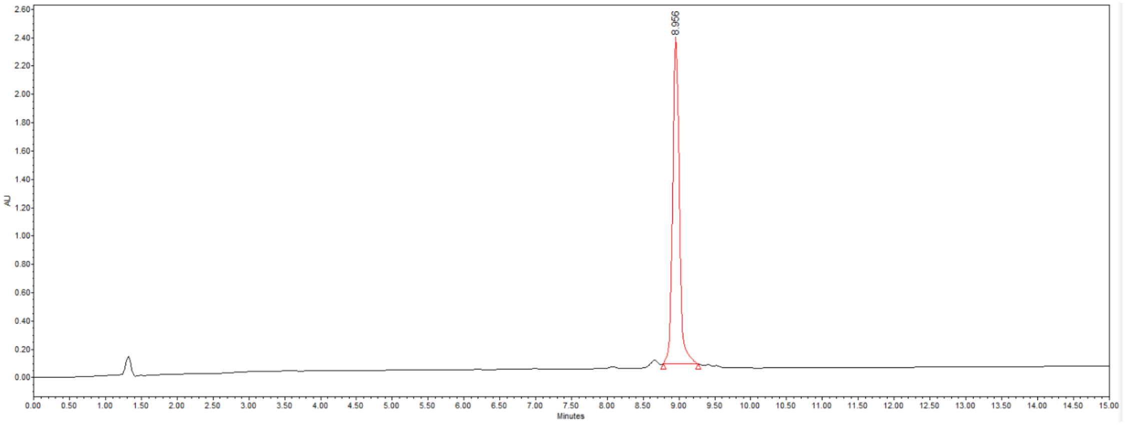

Figure S16: RP-HPLC of compound **16**.

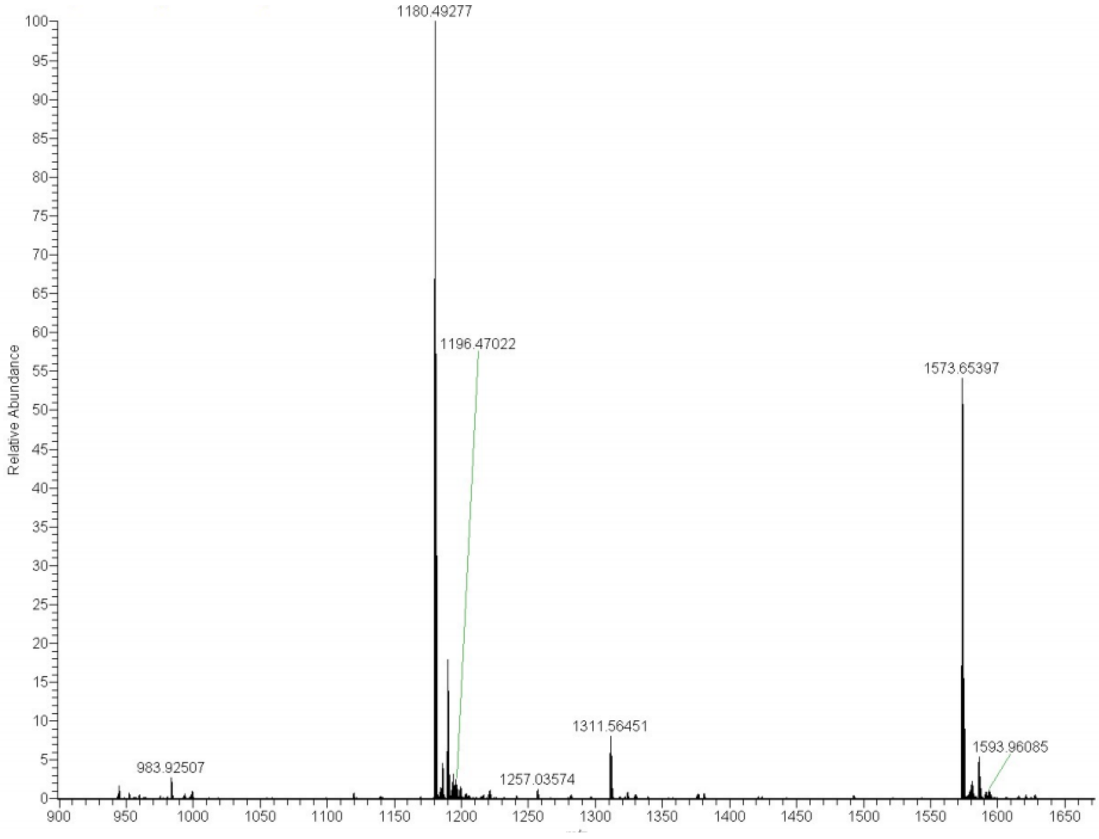

Figure S17: HRMS (ESI^{+}-TOF) of compound **16**.

Compound 18

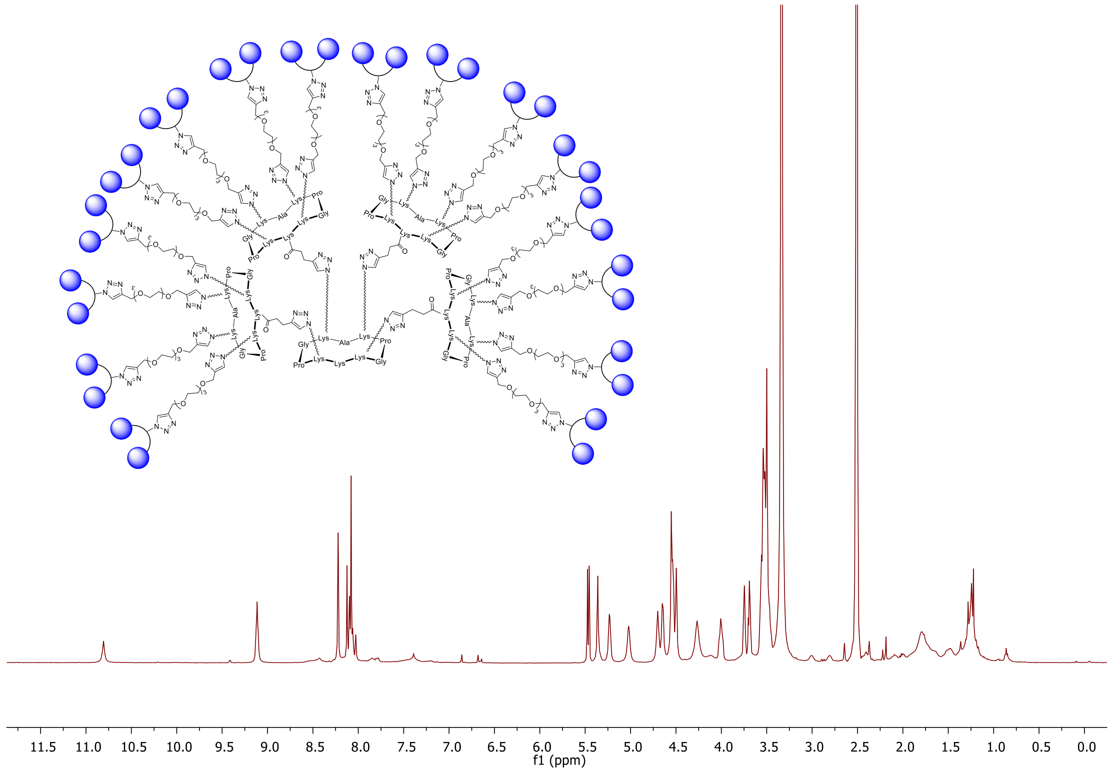

Figure S18: ^{1}H NMR spectra of compound **18** (DMSO, 500 MHz, 298 K).

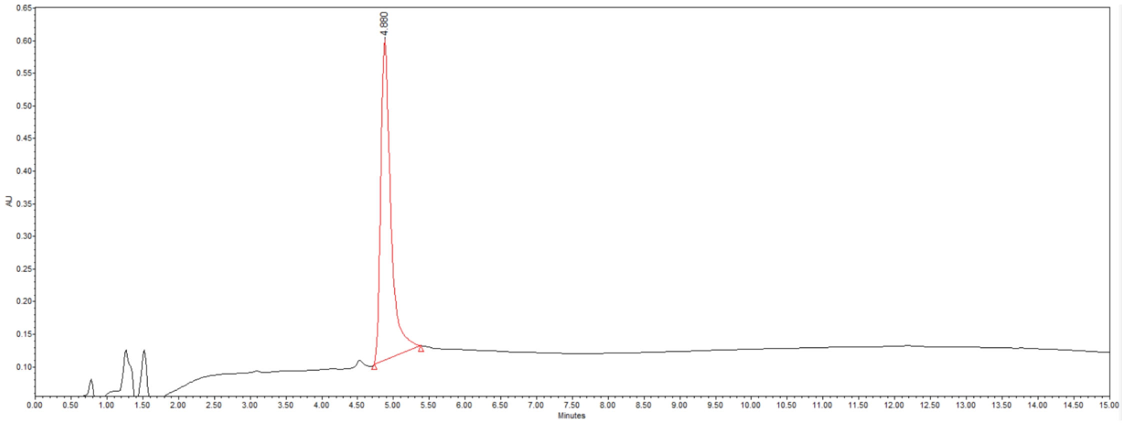

Figure S19: RP-HPLC of compound **18**.

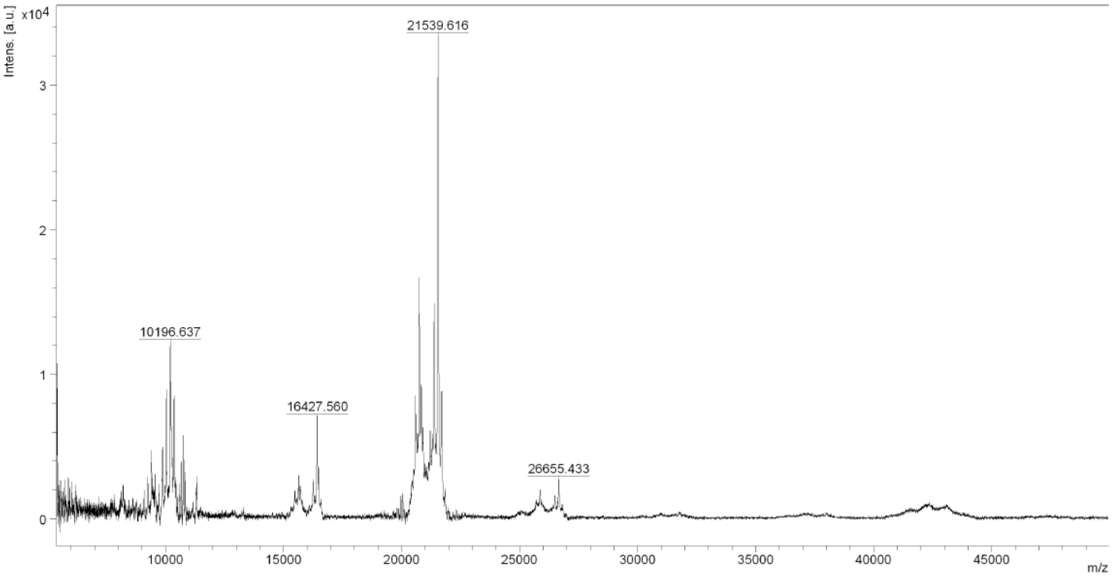

Figure S20: MALDI-TOF of compound **18**.

Compound 19

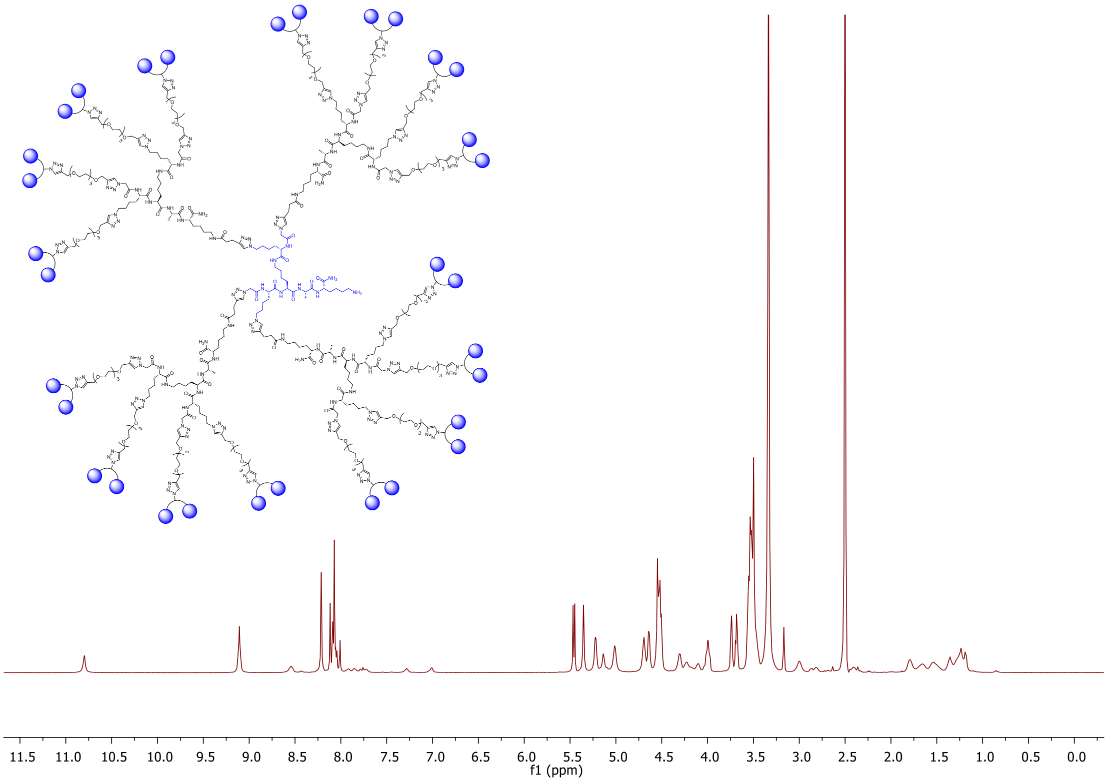

Figure S21: ^{1}H NMR spectra of compound **19** (DMSO, 500 MHz, 298 K).

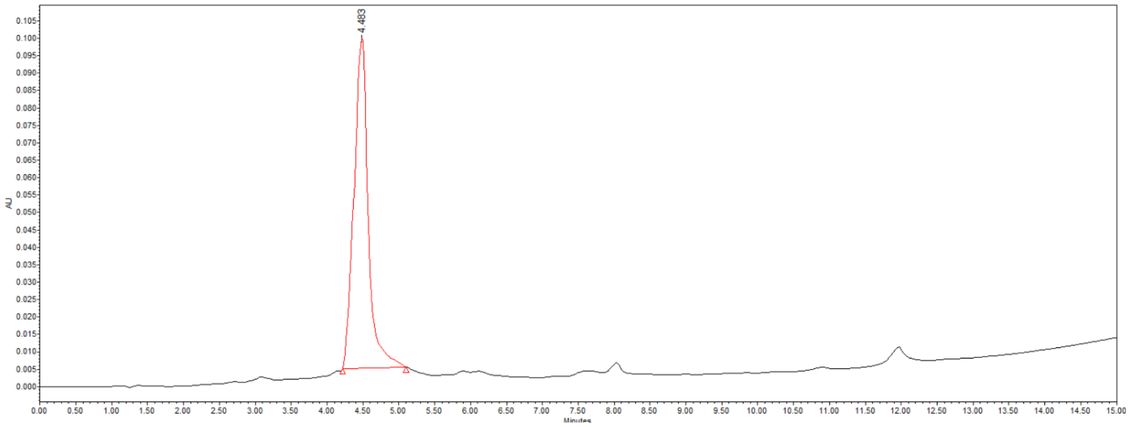

Figure S22: RP-HPLC of compound **19**.

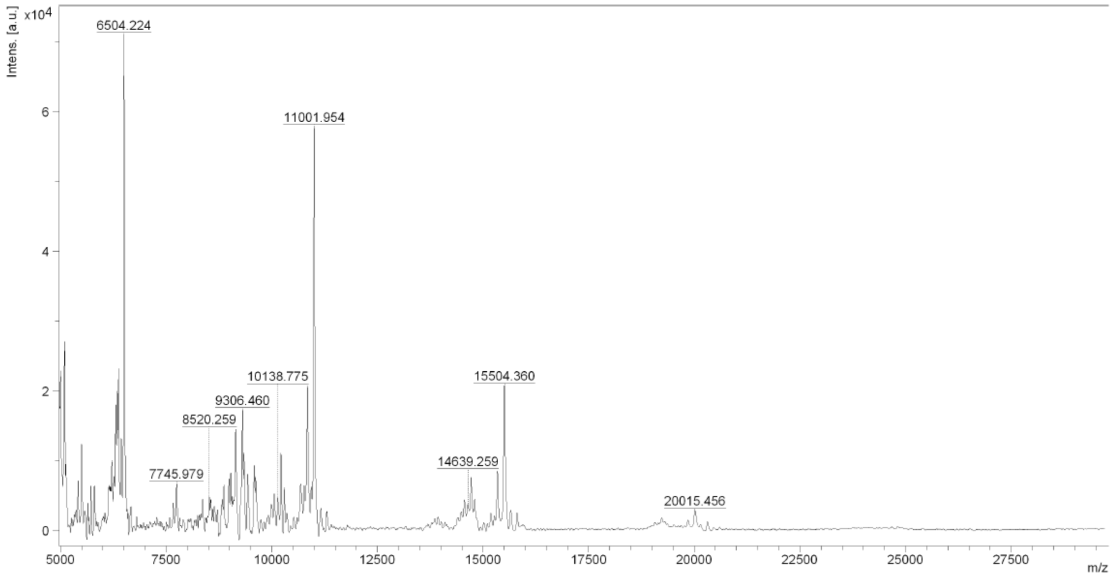

Figure S23: MALDI-TOF of compound **19**.

Table S1: Shows the molecular weights and concentration (M) corresponding to 1 mg/mL used in the adhesion assays

Cmpd	MW (g/mol)	Conc @ 1 mg/ml
**1**	721	1.38 mM
**11**	5027.99	0.199 mM
**14**	7709.52	0.129 mM
**16**	4722.62	0.212 mM
**18**	21538.42	0.0464 mM/46.4 µM
**19**	20014.58	0.05 mM/50 µM

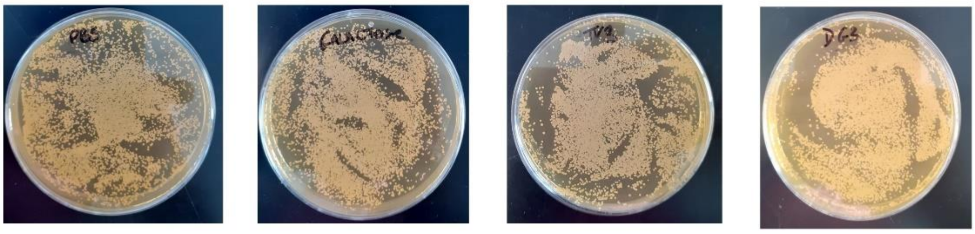

Control: PBS **D-Galatose** **1** **16**

Figure S24: Images of *C. albicans* after being incubated in the presence of each compound and grown on YEPD agar plates.
